# Differential expression of gene co-expression networks related to the mTOR signaling pathway in bipolar disorder

**DOI:** 10.1038/s41398-022-01944-8

**Published:** 2022-05-04

**Authors:** Sung Woo Park, Mi Kyoung Seo, Maree J. Webster, Jung Goo Lee, Sanghyeon Kim

**Affiliations:** 1 Department of Convergence Biomedical Science, College of Medicine, Inje University, 75 Bokji-ro, Busnajin-gu, Busan, 47392 Republic of Korea; 2 Paik Institute for Clinical Research, Inje University, 75 Bokji-ro, Busnajin-gu, Busan, 47392 Republic of Korea; 3 Stanley Brain Research Laboratory, Stanley Medical Research Institute, 9800 Medical Center Drive, Rockville, MD 20850 USA; 4 Department of Psychiatry, College of Medicine, Haeundae Paik Hospital, Inje University, 875 Haeun-daero, Haeundae-gu, Busan, 47227 Republic of Korea

**Keywords:** Bipolar disorder, Molecular neuroscience

## Abstract

Bipolar disorder (BPD) is a severe mental illness characterized by episodes of depression and mania. To investigate the molecular mechanisms underlying the pathophysiology of bipolar disorder, we performed transcriptome studies using RNA-seq data from the prefrontal cortex (PFC) of individuals with BPD and matched controls, as well as data from cell culture and animal model studies. We found 879 differentially expressed genes that were also replicated in an independent cohort of post-mortem samples. Genes involving the mechanistic target of rapamycine (mTOR) pathway were down-regulated, while genes interrelated with the mTOR pathway such as Janus kinase (JAK)-signal transducer and activator of transcription (STAT) pathway were up-regulated. Gene co-expression network analyses identified a module related to the mTOR pathway that was up-regulated in BPD and also enriched for markers of endothelial cells. We also found a down-regulated co-expression module enriched for genes involved in mTOR signalling and in mTOR related pathways and enriched with neuronal markers. The mTOR related modules were also replicated in the independent cohort of samples. To investigate whether the expression of the modules related to mTOR signalling pathway could be differentially regulated in different cell types we performed comparative network analyses in experimental models. We found both up-regulated modules in the PFC significantly overlapped with an up-regulated module in the brain endothelial cells from mice treated with lipopolysaccharides (LPS) and mTOR related pathways such as JAK-STAT, PI3K-Akt and ribosome were enriched in the common genes. In addition, the down-regulated module in the PFC significantly overlapped with a down-regulated module from neurons treated with the mTOR inhibitor, Torin1 and mTOR signalling, autophagy, and synaptic vesicle cycles were significantly enriched in the common genes. These results suggest that co-expression networks related to mTOR signalling pathways may be up- or down-regulated in different cell types in the PFC of BPD. These results provide novel insights into the molecular mechanisms underlying the pathophysiology of BPD.

## Introduction

Bipolar disorder (BPD) is a serious, chronic mental disorder with high heritability [1]. BPD is characterized by extreme changes in mood ranging from extremely elated and energized manic episodes to extremely sad and indifferent depressive episodes [2]. The lifetime morbid risk and 12-month prevalence of BPD are known to be about 4.1% and 1.8% in the Unites States respectively [3]. BPD is a complex disorder believed to be caused by a complex interaction between multiple genetic and various environmental factors that modify gene expression and impact the molecular mechanisms that underlie the disorder

Transcriptome analysis including gene co-expression network analysis has been used to investigate the pathophysiology underlying psychiatric disorders including BPD at a transcriptional level. Previous transcriptome analyses of postmortem brain tissues have implicated genes and pathways related to synaptic function, energy metabolism, autophagy, neuroplasticity and immune/inflammation response in the aetiology of this disorder [4–8]. Genes involved in the ubiquitin cycle and synaptic function are downregulated [4] while those involved in neuroplasticity are also differentially expressed in the PFC of individuals with BPD [5]. In addition, co-expression modules related to immune function [6], glial and neural cell genesis, glial cell differentiation [7] and the MAPK signalling pathway [8] are differentially expressed in the brain of individuals with BPD as compared to controls. The mTOR signalling pathway has been implicated in all these processes and also in the aetiology of psychiatric disorders [9].

The mechanistic target of rapamycine (mTOR) is a serine/threonine-protein kinase and is an important subunit of the mTOR complex 1 (mTORC1) and mTOR complex 2 (mTORC2) [10]. mTOR is involved in metabolism, protein synthesis and autophagy to regulate cell growth and achieve anabolic effects [11]. In the central nervous system, when mTOR signalling is activated, the synthesis of synaptic proteins is increased and autophagy is suppressed, promoting normal nerve growth and synapse formation [12]. The mTOR signalling pathway also plays a key role in neuronal cell growth, proliferation and synaptic transmission [13–15]. Over-activation of mTOR signalling increases neuronal growth and synaptic transmission in excitatory, as well as GABAergic neurons, while an mTOR inhibitor, rapamycin decreases synaptic transmission in glutamatergic synapses [14]. Moreover, in cultured glutamatergic hippocampal neurons, genetically inactivated mTORC1 affects postsynaptic transmission, whereas inactivated TORC2 affects presynaptic transmission [15]. Given the critical role of the mTOR pathway, it has been considered a key signalling pathway abnormally regulated in BPD [16]. Previous studies have focused on the translational control of the mTOR signalling pathway on synaptic function, while the transcriptional control of the signalling pathway is less well studied.

In this study, we performed differential gene expression analysis and gene co-expression analysis using RNA-seq data from two independent cohorts of human postmortem brain samples to identify the intracellular signaling pathways, particularly mTOR and interrelated pathways that may be implicated in individuals with BPD. We then investigated whether the results from these studies could be validated using experimental systems of cell culture and a mouse model.

## Methods

### RNA-Seq data from human post-mortem samples

#### Discovery study

The RNA-Seq data was generated from the PFC of 63 individuals with BPD and 68 unaffected controls from 3 Stanley Medical Research Institute (SMRI) tissue collections; the Neuropathology Consortium (NPC), the Array Collection (AC) and the New Stanley Collection (NSC). To increase the sample size of the study, we also combined the publicly available RNA-Seq data from an additional 29 unaffected controls from the NCBI short read archive database (https://www.ncbi.nlm.nih.gov/sra) as describe previously [17]. Two unaffected controls from the SMRI tissue collections were excluded from downstream analysis because they had autoimmune-related conditions. Two additional outlier samples were removed because sample RIN was lower than 5.0 or PMI was longer than 91 h. We conducted a clustering analysis on the adjusted RNA-Seq data to detect outlier data and two more samples were excluded from the network analysis. The final network analysis included RNA-Seq data from the PFC of 60 individuals with BPD and 94 unaffected controls (Supplementary Table 1).

#### Replication study

RNA-seq data, derived from the PFC of 260 individuals from the NIMH HBCC brain bank (dbGAP database accession number phs000979.v2.p2), was utilized for the replication study. Consistent with the methods used in the discovery study, low quality FASTQ data were excluded. To establish a cohort with demographic data comparable to that for the discovery study we excluded samples if age of death was younger than 20 or older than 63 years, and if PMI was longer than 70 h. Thus, RNA-Seq data from the PFC of 55 individuals with BPD and 122 unaffected controls were used for the replication study. (Supplementary Table 1).

### Primary cortical cell cultures

All animal procedures were performed in accordance with guidelines of the Institutional Animal Care and Use Committee (IACUC), Inje University, Republic of Korea, and approved by IACUC at the College of Medicine of Inje University (approval no. 2016–044). One or two embryonic day 19 Sprague-Dawley rats were used for each primary cortical cell culture, and a total of 6 embryonic day 19 Sprague-Dawley rats (Orient Bio, Seongnam, Korea) were used in this study. Cortices were dissected and dissociated in neurobasal medium (Gibco, Thermo Fisher Scientific, Waltham, MA, USA) with 0.03% trypsin (Gibco) for 20 min. The neurons were seeded randomly at a density 2 × 10^5^ cells per 6-well plate for RNA-Seq and Western blot analysis and 1 × 10^4^ cells per 96-well plate for MTT assay. No blinding was required for group assignments in this study. Cortical neurons were maintained in neurobasal medium with 1% fetal bovine serum (FBS; Gibco), 1% horse serum (Gibco), 2% serum-free B-27 supplement (Gibco), 0.25% l-glutamine (Gibco), and 50 U/mL penicillin–streptomycin (Gibco) at 37°C, 5% CO_2_, and 95% humidity. After incubation for 10 days, the cells were cultured with Torin 1 (mTOR inhibitor; Calbiochem, La. Jolla, CA, USA) or LY2584702 (S6K inhibitor; Selleckchem, Houston, Texas, USA) for 24 h before being harvested for further analysis.

#### Drug treatment

Ten mM of the mTOR inhibitors, Torin 1 and the S6K inhibitor, LY2584702, were dissolved in dimethyl sulfoxide (DMSO) and diluted to various concentrations (final concentration of 1% DMSO) with neurobasal medium. For dose response, cultured neurons were incubated with concentrations of Torin 1 or LY2584702 ranging from 10 nM to 2 µM. Cells were collected after 24 h of drug treatment for the cell viability assay [3-[4,5-dimethylthiazol-2-yl]-2,5-diphenyltetrazolium bromide (MTT) (Supplementary Methods; Supplementary Fig. 1) and for Western blot analysis (Supplementary Methods). Proteins predicted to be dysregulated by Torin 1 treatment of primary neuronal cells included phospho-mTORC1, phospho-Akt, phospho-S6K, phospho-S6, PSD-95, GluA1, Beclin 1, and LC3B (Supplementary Methods; Supplementary Figs. 1 and 2). Dendritic outgrowth and spine density were measured to determine the effect of Torin1 on neuronal growth and structure (Supplementary Methods). After establishing the optimal dose, cells were treated with 250 nM of Torin 1 or LY2584702 and collected 24 h following treatment for analysis of gene expression.

### RNA-Seq of cultured primary neuronal cells

RNA samples were extracted from 12 Torin1-treated primary neurons and 12 mock-treated neurons using Trizol kit (Invitrogen) following the manufacturer’s instruction. The isolated RNA samples were stored at -80 degree. RNA samples were submitted to Macrogen (Seoul, Korea) for RNA sequencing.

cDNA libraries were prepared from 1 microgram of RNA per sample using Illumina TruSeq RNA Sample Preparation v2 kit following the manufacturer’s instructions. Sequencing libraries were created and sequenced with Illumina platform. In brief, samples were tested for quality with the Agilent Bioanalyzer chip before Illumina adapters were added. Fifteen cycles of PCR were used to amplify the cDNA library with Illumina adaptors. qPCR was also performed for quantity check after the Illumina cDNA libraries were prepared. The libraries were multiplexed and loaded on a flow cell for cluster generation on cBot (Illumina). The Illumina Real-Time Analysis (RTA) module was used to perform image analysis and base calling and the BCL Converter (CASAVA v1.8.2) was used to generate the sequence reads (FASTQ files). Sequencing depth was over 70 million (2× 100-bp 35 million paired-end) sequencing reads.

### RNA-Seq data from cerebral endothelial cells of LPS treated mice

Publicly available RNA-seq data derived from the brain endothelial cells of mice treated with LPS for 2 h and control mice (*n* = 3 each group) were downloaded from the SRA database (accession number SRR274654) [18] and used for validation of the human postmortem results. Briefly, mice were administrated a single intraperitoneal injection of LPS or phosphate-buffered saline and sacrificed at 2 h postinjections. Cells were dissociated from the brains of mice and then cerebral endothelial cells (CD45- CD13- CD31 + ) were sorted using florescence-activated cell sorting.

### Quality control and analysis of RNA-Seq data

Quality control of the raw FASTQ files, mapping the RNA-Seq reads and quantifying the mapped reads were performed as previously described with some modifications [17]. Briefly, the raw data from all samples passed the initial quality control using FASTQC (https://www.bioinformatics.babraham.ac.uk/projects/fastqc/).

All human reads were then mapped to the GRCh38 human reference genome using HISAT2 [19]. Sequencing reads from rat primary neurons and the endothelial cells of LPS treated mice were mapped to Ensembl rat reference genome (Rnor 6.0.97) and mouse reference genome (mm10) respectively. Counting of the mapped reads was performed by htseq-count (subprogram of HTSeq), no strand-specific option, and intersection-nonempty option.

#### Differential gene expression analysis

To identify differentially expressed genes in the PFC between BPD and controls and between the experimental treatment groups and controls, we first identified the potential confounding variables in the RNA-Seq data using surrogate variable analysis (SVA) [20]. We then compared read counts of genes from BPD and controls or between the treatment groups and their controls after adjusting for potential confounding variables using generalized linear model methods in the EdgeR software [21]. The software estimates the dispersion using the Cox-Reid profile-adjusted likelihood method. False discovery rate (FDR) less than 0.05 was considered significant.

#### Gene co-expression network analysis

Gene co-expression modules were generated from the RNA-Seq data by weighted gene co-expression network analysis (WGCNA) R package [22], as previously described [17]. First, we identified and adjusted for the potential confounding variables in the RNA-Seq data using surrogate variable analysis (SVA) [20]. The standardized residuals from the linear regression including the surrogate variables were used to generate gene co-expression networks using WGCNA [22]. To construct a weighted co-expression network we selected the power for scale-free topology fitting index (R^2^) is ≥0.9 [23]. Correlation analyses were performed between co-expression modules and traits such as diagnosis, treatment of drug, RIN and other descriptive variables to identify modules that were associated with the trait of interest and/or confounding factors. To adjust for multiple testing when we performed the correlation analyses, we used the MPTCorr.r package (http://www.psych.umn.edu/faculty/waller/downloads/mpt/mptcorr.r) [24]. Adjusted P-values less than 0.05 were considered significant. If a module eigengen value was significantly different between case and control or treatment and non-treatment, the module was defined as an associated module. If an associated module was significantly enriched with differentially expressed genes, the module was defined as a differentially expressed module. To evaluate whether each co-expression module was significantly enriched for differentially expressed genes, we performed Fisher’s exact test. The network connections were visualized using Cytoscape [25]. The consensus co-expression modules from two studies were identified as previously described [26]. *P* values of significance for each of the pairwise overlaps were obtained by permutation tests in R. Since we performed 1 million permutations, the lowest possible P-value we can obtain from our analysis is 1e-06. If *P* values were less than 1e-06, we performed a Fisher’s exact test using R script (https://bioconductor.org/packages/release/bioc/html/GeneOverlap.html). If the total number of all module genes is *n*, the number of module genes in A is *a*, and the number of differentially expressed genes, cell type-specific marker genes or another module genes in B is *b*. If the intersection between A and B is *t*, R script for Fisher’s exact test is “matrix(c(n-union(A,B), setdiff(A,B), setdiff(B,A), intersect (A,B)), nrow = 2)”.

#### Cell type enrichment analysis

To identify cell-type-specific marker genes enriched in each co-expression module, we performed a Fisher’s exact test using marker genes derived from human brain cells. To obtain specific marker genes for each cell-type, we first downloaded from a previous study [27], the top 1000 genes with three measures of the different brain cell-type relative expression. The three measures included absolute expression, enrichment, and specificity. The data was generated using 5 human cell-type-specific transcriptome-wide RNA expression data sets further validated using an orthogonal ATAC-seq dataset [27]. The 5 brain cell types were astrocyte (Ast), endothelial cell (EC), microglia (MG), neuron (Neu) and oligodendrocyte (OD). We compared the nodes (genes) in each of our brain-derived modules to the list of specific marker genes for each cell-type and evaluated the significance of enrichment between the gene lists.

#### Predicting target genes of transcription factors related to mTOR pathway

Target genes of transcription factors in the differentially expressed modules were predicted by plugin iRegulon [28] of Cytoscape program [25]. The NEScore >3 and FDR on motif similarity <0.001 were set as thresholds. Significantly, enriched biological processes in the transcription factor-target gene network were identified by gProfiler [29]. FDR less than 0.05 was considered significant.

#### Functional annotation

Enrichment of KEGG pathways in the genes in the differentially expressed genes, co-expression modules or the consensus module were identify by gProfiler [29].

Fold enrichment score represents the ratio between list hits by list total and population hits by population total. For example, if 10% of a modules genes are involved in a specific pathway and 1% of genes in the total genome are involved in the same pathway, then the fold enrichment score is 10. FDR was computed using the Benjamini and Hochberg method to correct the error rate of multiple testing. FDR less than 0.05 was considered significant.

## Results

### Differential gene expression in PFC of individuals with BPD

We analysed RNA-Seq data from PFC of 60 subjects with BPD and 94 unaffected controls from the three SMRI tissue collections and other publicly available data (Supplementary Table 1). The expression of 2475 genes were upregulated and 1463 genes were down-regulated and in the PFC of individuals with BPD at FDR *q* < 0.05. Three KEGG pathways including ribosome were significantly enriched in the up-regulated genes in the PFC of individuals with BPD (Supplementary Table 2). Ribosome was the most significant enriched pathway (FDR = 4.4e-19) with 67 genes belonging to this pathway. In contrast, 15 KEGG pathways including GABAergic synapse, ubiquitin-mediated proteolysis, glutamatergic synapse and autophagy were significantly enriched in the down-regulated genes (Supplementary Table 3). Several of the 15 KEGG pathways, including ubiquitin-mediated proteolysis and autophagy, are related to the mTOR pathway. While 18 genes involved in the mTOR pathway were differentially expressed (Supplementary Table 4), the mTOR pathway itself was not significantly enriched in the differentially expressed genes. Nevertheless within the 18 genes, some for example, EIF4EBP1 (eukaryotic translation initiation factor 4E) were up-regulated while others e.g. AKT3 (AKT serine/threonine kinase 3) were down-regulated, in individuals with BPD, indicating that genes involved in mTOR and related pathways are differentially expressed in BPD.

### Gene co-expression modules differentially expressed between BPD and controls

**Fig. 1:**
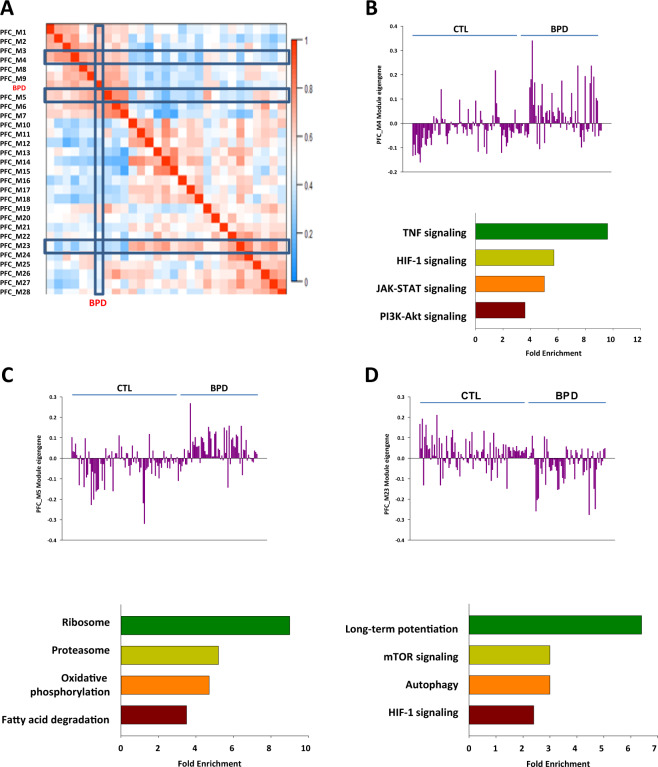
Co-expression modules differentially expressed between BPD and controls in PFC. The eigengene adjacency heatmap of the modules (**A**), the eigengene values across samples and major KEGG pathways significantly enriched in the genes in the PFC_M4 module (**B**), the PFC_M5 module (**C**) and the PFC_M23 module (**D**).

Co-expression network analysis as conducted to further investigate whether mTOR signalling and related pathways are differentially expressed in BPD. We conducted an unsupervised co-expression network analyses using the RNA-seq data adjusted for potential confounding variables. Twenty-eight co-expression modules were generated with the data and 17 modules were significantly associated with BPD (Fig.1A, Supplementary Table 5). Eight modules that significantly enriched for the differentially expressed genes were up-regulated in the PFC of BPD (Supplementary Table 6). Of the eight modules, only two (PFC_M4 and PFC_M5) were significantly enriched with a substantial number of pathways in the module genes. In the PFC_M4 module, TNF signaling pathway, HIF-1 signaling pathway, JAK-STAT signaling pathway and PI3K-Akt pathway were significantly enriched (Fig. 1B, Supplementary Table 7). In the PFC_M5 module, ribosome, proteasome, and fatty acid degradation were the major significant KEGG pathways (Fig. 1C, Supplementary Table 7).

Of seven down-regulated modules, the PFC_M23 module was the most significantly down-regulated and enriched for a number of KEGG pathways in the module genes (Fig.1D, Supplementary Table 7). Consistent with previous results from multiple gene expression profiling studies including a study using a subset of these cases [30] genes related to synaptic vesicle cycle and dopaminergic, GABAergic, glutamatergic and serotonergic synapses were significantly enriched in the down-regulated PFC_M23 module (Supplementary Table 7). We also found the mTOR signaling pathway (Supplementary Table 8) and several signaling pathways downstream of mTOR, such as autophagy and HIF-1 significantly enriched in this module (Fig.1D).

### Cell type enrichment

We next compared the genes of the modules to the list of cell-type-specific marker genes derived from 5 different brain cells from human cortex to identify specific cell types that may be associated with the modules. The up-regulated PFC_M4 module was highly enriched with genes for markers specific to endothelial cells (*P* = 1.2e-14) (Supplementary Table 9). Interestingly, mTOR is involved in the four signaling pathways enriched in the PFC_M4 module, e.g. PI3K-Akt signaling pathway can activate mTOR sigaling by direct phosphorylation as well as inhibition of Tuberous Sclerosis Complex 2 (TSC2) which is an inhibitor of the mTOR pathway [31]. JAK-STAT signaling can regulate the mTOR signaling pathway [32] and the HIF-1 signaling pathway, and proteasome and fatty acid degradation are known to interact with mTOR signaling [33]. Thus, mTOR signaling appears to be up-regulated in the endothelial cells of the PFC in BPD.

While there were no cell-specific markers significantly enriched in the PFC_M5 module, it was enriched for the KEGG pathways ribosome and metabolism, which are regulated by the mTOR pathway [34, 35]. Thus, the mTOR signalling pathway may also be up-regulated in other cells of the PFC in BPD.

The down-regulated PFC_M23 module, was significantly enriched for neuronal markers (*P* < 2.2e-16) (Supplementary Table 9). Interestingly, the mTOR signaling pathway and several signaling pathways downstream of mTOR, such as autophagy and HIF-1 signaling were also enriched in the PFC_M23 module. This suggests that the mTOR signaling pathway may be differentially regulated within the different cell types i.e. up-regulated in the endothelial and down-regulated in the neurons of the PFC in BPD.

We also examined whether antidepressant and/or mood stabilizer medications effect mTOR activation. We examined the associations between module eigengenes and lithium, other mood stabilizers and antidepressants. There were no significant associations between any modules and any of these treatments (Supplementary Table 5).

### Transcription factors of the co-expression networks

**Fig. 2:**
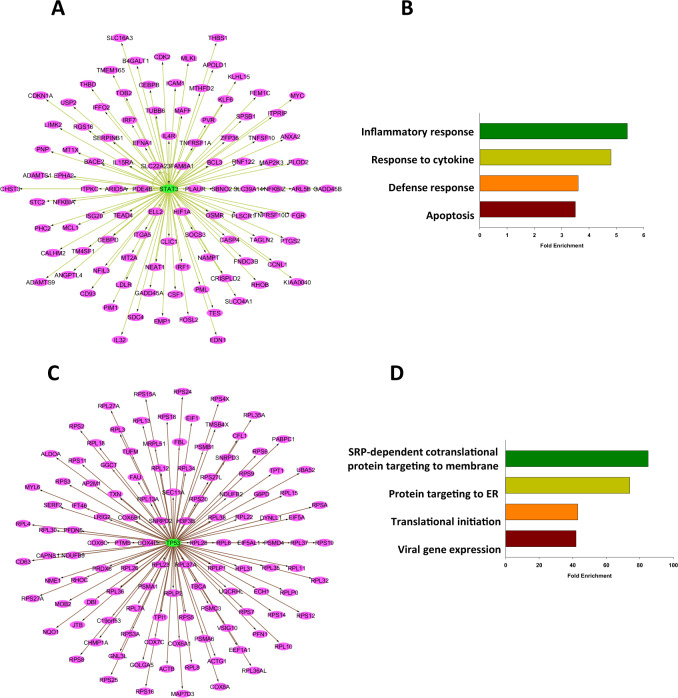
Transcription factors and their target genes in the PFC_M4 and the PFC_M5 module. Visualization of STAT3-target gene network in the PFC_M4 module (**A**), Biological processes significantly enriched in the network (**B**). Visualization of p53 -target gene network in the PFC_M5 module (**C**) and biological processes significantly enriched in the network (**D**).

Our co-expression network results indicate that mTOR related modules appear to be differentially regulated in different cell types in the PFC of individuals with BPD compared to unaffected controls. mTORC1 regulates gene expression through several specific transcription factors including STAT3 [36], NFAT5 [37], FOXO [38] and p53 [39]. Using iRegulon software we identified the target genes (regulons) of the specific transcriptional factors that are regulated by mTORC1 in the nodes (genes) of the significant modules. In the PFC_M4 module we identified 103 direct target genes of STAT3 (64% of the module genes; NES = 5.2). Moreover, immune/inflammatory response and apoptosis were the biological processes significantly enriched in the direct target genes (Fig. 2A, B, Supplementary Table 10). Thus, STAT3 may be a master regulator of the co-expression module that is associated with up-regulation of genes related to the immune/inflammatory responses in endothelial cells of the PFC in BPD. In the PFC_M5 module, we detected 117 target genes of p53 (NES = 6.3) which were significantly enriched for biological processes related to translation, such as SRP-dependent co-translational protein targeting to membrane, and translational initiation (Fig. 2C, D, Supplementary Table 11). We did not identify any transcription factors regulated by TORC1 in the genes of downregulated PFC_M23 module.

### Replication of the differentially expressed genes in the PFC in BPD using an independent data set

We replicated the differentially expressed genes in the PFC of BPD using an independent RNA-seq data set derived from the PFC of 55 individuals with BPD and 122 unaffected controls from the NIMH HBCC brain bank (Supplementary Table 1). The expression of 3399 genes were upregulated and 2139 genes were down-regulated in the PFC of individuals with BPD at FDR *q* < 0.05. Of the 3399 upregulated genes, 409 overlapped those up-regulated in the SMRI samples (Supplementary Table 12). KEGG pathways interrelated with mTOR signalling, such as PI3K-Akt signaling and JAK-STAT signalling pathways were enriched in the up-regulated genes (Supplementary Table 13). Of the 18 differentially expressed genes involved in the mTOR pathway that were found in the discovery study, 11 were replicated in the independent data set (Supplementary Table 14). We did not find up-regulation of ribosomal protein genes in the replication dataset.

Of the 2139 down-regulated genes, 470 overlapped those down-regulated in the SMRI samples (Supplementary Table 12). Eighteen KEGG pathways including the mTOR signalling pathway and autophagy were enriched in these commonly downregulated genes (Supplementary Table 15).

### Replication of the mTOR pathway-related modules

Five modules were differentially expressed in the PFC of BPD in the replication dataset (Supplementary Fig. 3A, Supplementary Tables 16, 17). When the nodes (genes) in the modules were compared to those in the mTOR related modules from the SMRI samples we found significant overlap of the co-expression modules between both data sets. The up-regulated and mTOR related module, PFC_M4, from the SMRI samples showed significant consensus with a module, R_PFC_M15, from the replication dataset (*P* < 2.2e-12) (Supplementary Fig. 3B). The KEGG pathways related to the mTOR pathway, such asTNF signalling, JAK-STAT signalling and PI3K-Akt signalling were significantly enriched in the genes common to both modules (Supplementary Fig. 3C, Supplementary Table 18). The other up-regulated module, PFC_M5, did not show consensus with any modules from the replication data. The down-regulated module, PFC_M23 showed significant consensus with a module, R_PFC_M7, from the replication dataset (*P* < 2.2e-12) (Supplementary Fig. 1B). The KEGG pathways significantly enriched for the genes common to both modules, included mTOR signaling and its possible downstream pathways such as autophagy (Supplementary Fig. 3D, Supplementary Table 19). Thus, the co-expression modules related to mTOR signalling and interrelated pathways in BPD are replicated in an independent cohort of PFC samples.

### Gene co-expression modules differentially regulated by the mTOR inhibitor Torin1 in primary neurons

Because our data indicates that mTOR signalling may be downregulated in neurons we determined whether inhibiting mTOR signalling in cultured neurons would result in similar co-expressed modules of gene expression as found in BPD subjects. We performed comparative network analysis between the modules from our human study and those from cultured neurons treated with the mTOR inhibitor Torin1.

**Fig. 3:**
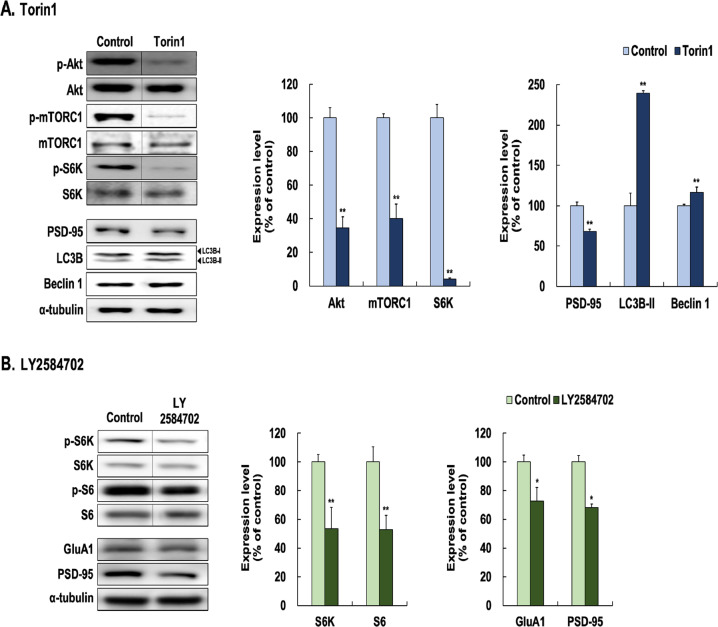
Effects of Torin1 and LY2584702 in primary neuronal cells. Neuronal cells were treated with Torin1 (**A**) and LY2584702 (**B**) for 24 h at 250 nM concentration. Cell lysates were analysed by SDS-PAGE and Western blot analyses with each of the primary antibodies. Western blot analyses revealed the levels of phospho-Ser473-Akt, phospho-Ser2448-mTORC1, phospho-Thr389-S6K, phospho-Ser240/244-S6, PSD-95, GluA1, LC3B-II, and Beclin 1.Representative images and quantitative analyses normalized to the total levels for each protein or α-tubulin are shown. The original images for crude blot are shown in Supplementary Fig. 4 (Torin1) and in Supplementary Fig. 6 (LY2584702). Values (*n* = 4–6) are shown as the mean ± SEM expressed as a percentage of the control cells (no drug) values. *p < 0.05, **p < 0.01, *unpaired Student t test*.

First, we determined, by Western blot if in fact Torin 1 treatment on primary neuronal cells resulted in dysregulation of the mTOR signaling pathway (Fig. 3A, Supplementary Fig. 4). Normally, mTOR activity would increase levels of phospho-Akt, phospho-mTORC1, phospho-S6K and PSD-95 on proteins of mTOR signaling and decrease LC3B-II and beclin 1 on autophagy-related proteins. As predicted we found mTOR inhibition with 250 nM of Torin1 significantly decreased phospho-Akt (66% of control, *t* = 7.278, *p* < 0.001), phospho-mTORC1 (60% of control, *t* = 6.706, *p* < 0.001), phospho-S6K (96% of control, *t* = 11.740, *p* < 0.001), and PSD-95 (32% of control, *t* = 6.103, *p* < 0.001). We also found Torin1 increased LC3B-II (136% of control, *t* = 8.618, *p* < 0.001) and beclin1 (17% of control, *t* = 2.684, *p* = 0.036) levels. Torin1 treatment also decreased dendritic outgrowth and spine density (Supplementary Fig. 5).

In addition, we also investigated the regulation of downstream target of mTORC1 signalling, phospho-S6K, which is inhibited by LY2584702 (Fig. 3B, Supplementary Fig. 6). We found 250 nM of LY2584702 significantly decreased the levels of phospho-S6K (43% of control, *t* = 4.900, *p* = 0.003), phospho-S6 (47% of control, *t* = 4.030, *p* = 0.007), GluA1 (27% of control, *t* = 2.530, *p* = 0.045), and PSD-95 (26% of control, *t* = 3.640, *p* = 0.011) in primary neuronal cultures.

We constructed 23 co-expression modules using the RNA-Seq data from the primary neurons treated with mock or Torin1 (Supplementary Table 20). Two of these co-expression modules, Torin1_M6 and Torin1_M15, were enriched for genes differentially expressed by Torin1 treatment in primary neurons (Supplementary Table 21).

Torin1_M15 was up-regulated by Torin1 treatment in the primary neurons, whereas Torin1_M6 was down-regulated. Torin1_M15 was significantly enriched for ErbB signalling, autophagy, Wnt signalling and metabolic pathway (Supplementary Table 22). The Torin1_M6 module was a large module consisting of 4769 nodes (genes) and significantly enriched for KEGG pathways related to proteasome, cell cycle, FoxO signaling and mTOR signaling (Supplementary Table 22). These results indicate that inhibition of the mTOR pathway by Torin 1 can downregulate a co-expression module that includes genes of the mTOR signaling pathway and related pathways in cultured primary neurons.

### Comparison of the modules differentially expressed in BPD to those from primary neurons treated with the mTOR inhibitor Torin1

**Fig. 4:**
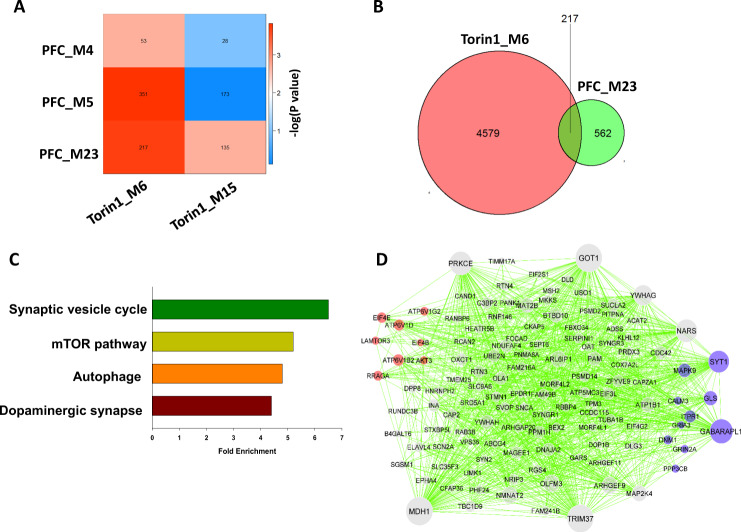
Comparison of the differentially expressed modules in the PFC of BPD to those from Torin1 treated neurons. Pairwise comparisons of the modules differentially expressed in BPD and the differentially expressed modules in Torin1-treated neurons (**A**). The color code of the heatmap encodes −log (*P*-value). The *P*-values were calculated by permutation test for the overlap of the two modules. The numbers in the heatmap indicate gene counts in the intersection of two modules. Venn diagram shows the number of common and unique genes between the PFC_M23 and the Torin1_M6 module (**B**). KEGG pathways significantly enriched in the genes common to both co-expression modules (**C**) and visualization of the network connections of the common genes between the two modules using Cytoscape [25] (**D**). The hub genes are larger circles in the network. Genes related to mTOR signaling pathway are in red and genes related to synaptic vesicle cycle are in blue.

To validate the differential expression of the co-expression modules related to the mTOR pathway in neurons from BPD, we compared the 3 BPD-associated co-expression modules to the Torin1_M6 and Torin1_M15 modules that were differentially regulated by Torin1 in the primary cultured neurons. The Torin1_M6 module significantly overlapped with two modules, PFC_M5 and PFC_23 that were differentially expressed in BPD, while the Torin1_M15 did not overlap with any differentially expressed modules (Fig. 4A). Pathways significantly enriched in the genes common to both PFC_M5 and Torin1_M6 included oxidative phosphorylation, proteasome, ribosome and spliceosome (Supplementary Table 23). A total of 217 nodes (genes) were common to the two downregulated modules PFC_M23 and the Torin1_M6 module (Fig. 4B). Synaptic vesicle cycle, mTOR pathway, autophage and dopaminergic synapse were significantly enriched in the genes common to both these downregulated modules (Fig. 4C, D, Supplementary Table 24). Eleven of the 217 common genes, including AKT3, ATP6V1B2, EIF4B, EIF4E, and LAMTOR3, are involved in the mTOR signaling pathway (Fig. 4D). These results support our finding that co-expression modules related to the mTOR signaling pathway can be downregulated in neurons from the PFC in BPD.

### Comparison of the BPD-associated modules to those from brain endothelial cells of mice treated with LPS

**Fig. 5:**
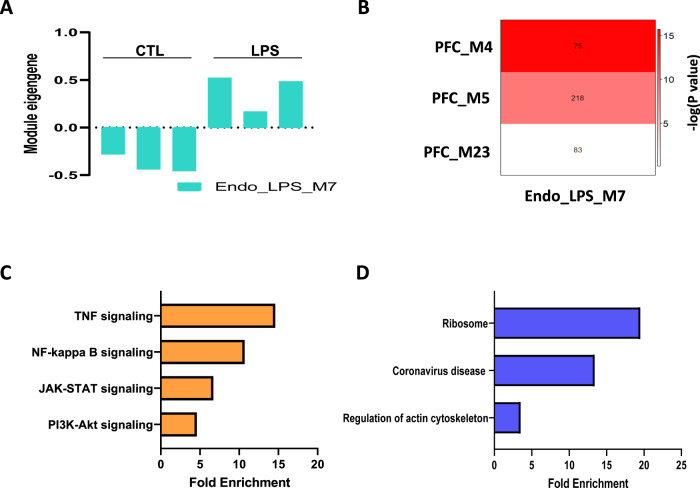
Comparison of differentially expressed modules in the PFC of BPD to that from cerebral endothelial cells of mice treated with LPS. **A** The eigengene values of a differentially expressed module, Endo_LPS_M7 in the endothelial cells of mice treated with LPS for 2 h. **B**, Pairwise comparisons of the modules differentially expressed in BPD and the differentially expressed module in the endothelial cells. The color code of the heatmap encodes −log (*P* value). The *P*-values were calculated by Fisher exact test for the overlapping of the two modules. The numbers in the heatmap indicate gene counts in the intersection of two modules. Major KEGG pathways significantly enriched in the genes common between PFC_M4 and Endo_LPS_M7 (**C**) and between PFC_M5 and Endo_LPS_M7 (**D**).

The co-expression network analysis from the human post-mortem study indicated that signalling pathways interrelated with the mTOR pathway may be up-regulated in the endothelial cells of the PFC in BPD and that pathways related to infection are also enriched in the module (Supplementary Table 7). To validate the upregulation of mTOR-related modules in the endothelial cells of BPD we compared the modules differentially expressed in BPD to the modules from endothelial cells from an infection mouse model. Using publicly available RNA-seq data from CD31 positive brain endothelial cells of mice treated with LPS and control mice [18], we constructed 8 co-expression modules and identified 1 module significantly upregulated by LPS (Fig. 5A, Supplementary Tables 25, 26). The nodes of Endo_M7 significantly overlapped with those of the upregulated PFC_M4 and PFC_M5 modules (Fig. 5B). Forty KEGG Pathways including TNF signalling, NF-kappa B signalling, JAK-STAT signalling and PI3K-Akt signalling were significantly enriched in the genes common to both PFC_M4 and Endo_LPS_M7 (Fig. 5C, Supplementary Table 27). Three KEGG pathways including ribosome and coronavirus disease were significantly enriched in the genes common to both PFC_M5 and Endo_LPS_M7 (Fig. 5D, Supplementary Table 28), indicating that both PFC_M4 and PFC_M5 may be derived from brain endothelial cells. These results supports our findings that signaling pathways interrelated with the mTOR pathway can be upregulated at the transcriptional level in the endothelial cells in BPD and that infections may be associated with the upregulated pathways.

## Discussion

We performed multiple transcriptomic studies that included human postmortem samples, cell culture, and a mouse model to explore the molecular mechanisms that may underlie BPD. We replicated the downregulated and one of two upregulated co-expression modules from the post-mortem study in a separate independent set of post-mortem data. We chose to focus on and further explore the association between BPD and the dysregulation of mTOR and related signaling pathways. In the human transcriptome study, a co-expression module enriched for mTOR-related pathways was up-regulated in the endothelial cells of BPD, whereas a module that also included the mTOR signalling pathway and related pathways was down-regulated in neurons. Moreover, using experimental systems we found similar modules of gene expression upregulated by LPS in endothelial cells and downregulated by mTOR inhibitors in neurons, thereby supporting the notion that similar or interrelated pathways can be differentially regulated in different cell types within the same tissue. We summarize our findings, the replicated data and the supporting data in Supplementary Table 29.

Genes involved in the PI3K-Akt, HIF-1, and FoxO signalling pathways as well as the endothelial cell markers were significantly co-enriched in the PFC_M4 module up-regulated in the BPD. Moreover, genes related to various infections and immune response pathways were also enriched in this module. These results suggest that activation of the mTOR signalling pathway may be associated with the immune/inflammation response in the endothelial cells of the PFC in individuals with BPD. The significant overlap between the PFC_M4 module and the up-regulated module from the brain endothelial cells of the LPS-treated mice adds support for the finding. A study of blood mononuclear cells showed altered Akt phosphorylation in BPD [40] and post-mortem studies report increased expression of genes related to inflammatory response in the PFC of BPD as compared to controls [41]. Protein and mRNA levels of IL1B, IL-1R and Nf-kB are increased in the PFC of BPD [42] and a previous large scale RNA-seq study showed significant up-regulation of the inflammatory Nf-kB signaling module in the PFC of BPD [43]. Consistent with these studies, we identified both the TNF signaling pathway and the NF-kB signaling pathway as significantly enriched in the PFC_M4 module. In addition, genes included in these signalling pathways were enriched in the genes common to the upregulated human PFC_M4 module and the up-regulated Endo_LPS_M7 module from brain endothelial cells of LPS-treated mice. While up-regulation of the genes related to immune/inflammation response in the brain of individuals with BPD has been reported previously, little is known about the possible underlying mechanisms. Our data suggest that the mTOR signaling pathway may play a key role in the up-regulation of genes related to the immune/inflammation response in the brain endothelial cells in BPD. Our data also suggests that the transcription factor, STAT3 acts as a master regulator of this module. mTORC1 can activate STAT3 by phosphorylation at Ser727 [44, 45]. Phosphorylation of Ser727 on STAT3 activates transcription of target genes by recruiting transcription co-factors [46]. A previous study shows microglia-specific STAT3 is associated with depression-like behaviours in mice [47]. Thus, the genes related to immune/inflammation response may be up-regulated in endothelial cells and/or activated microglia under the control of STAT3, and associated with BPD.

The up-regulated PFC_M5 module in BPD was enriched for the genes of ribosomal proteins, validating a previous gene expression study done using a subset of these same brain samples [30]. Ribosome biogenesis is essential for cell survival and growth [48]. The process is regulated by the mTOR pathway at multiple steps including the transcription of ribosomal proteins [35]. While the PFC_M5 module was not significantly enriched for any specific cell-type marker tested in this study, the module did significantly overlap with the up-regulated Endo_LPS_M7 module from brain endothelial cells of LPS-treated mice. The ribosomal protein genes were significantly enriched in the common genes. In addition, the module also overlapped with the Torin1_M6 module that was downregulated by inhibition of mTOR in primary neurons. Ribosomal protein genes were also significantly enriched in the common genes. Inhibition of mTORC1 induces down-regulation of RNA levels of genes encoding ribosomal proteins in primary human trophoblast cells [35]. These results suggest that similar co-expression modules including genes of ribosomal protein were regulated by mTOR regardless of cell types. The module was down-regulated by mTOR inhibition in neurons, while the module was up-regulated by LPS in brain endothelial cells. However, the consensus between the PFC_M5 and the Endo_LPS_M7 (*P* < 2.2e-16) is much more significant than that between the PFC_M5 and the Torin1_M6 (*P* = 2.0e-4), suggesting the PFC_M5 is more likely derived from brain endothelial cells in BPD.

We show that RNA levels of genes related to various synaptic functions, including dopaminergic, glutamatergic and GABAergic synapses were downregulated in BPD and KEGG pathways associated with these synaptic pathways were enriched in the downregulated, neuron related PFC_23 module. Moreover, these synaptic-related genes are co-regulated with the mTOR pathway in PFC_23, raising the possibility that mTOR signaling may be associated with synaptic function at a transcriptional level. Synaptic dysfunction has been implicated in the pathophysiology of both major depression and BPD [49]. In addition, decreased mTORC1 activity is associated with the pathophysiology of depression, and increasing mTORC1 activity by ketamine, an NMDA antagonist, has a rapid antidepressant effect [50]. Our results suggest that an inactivated mTOR pathway in the neurons may contribute to BPD by down-regulating genes related to synaptic function.

We identified 11 genes involved in the mTOR signaling pathway, including AKT3, ATP6V1B2, EIF4B, EIF4E, and LAMTOR3 that are common to the downregulated PFC_M23 neuronal module and the Torin1_M6 module downregulated by inhibition of mTOR in neuron culture. A previous study showed genes involved in the mTOR pathway including AKT1, mTOR, and BAD are down-regulated in the blood of drug naïve, BPD patients [16], which concurs with our results. We also identified nine genes associated with autophagy that are common to both the downregulated PFC_M23 module and the Torin1_M6 module from Torin1 treated neurons. Autophagy is a highly conserved biological process that induces degradation of dysfunctional cellular organelles and proteins in the lysosome [51]. Autophagy plays a critical role in the homeostasis of neurons and is implicated in psychiatric diseases including mood disorders [52]. A previous study using a subset of cases from this study (the AC collection) found that genes related to lysosomal function were differentially expressed in the anterior cingulate cortex between BPD and controls [53]. Our data suggest that the autophagy process may be associated with mTOR signaling pathways in the neurons of BPD at a transcriptional level. mTORC1 negatively regulates autophagy by phosphorylation of proteins involved in various steps of the process such as autophagy nucleation, autophagosome elongation, and autophagosome maturation [54–56]. Moreover, the mTOR pathway can regulate autophagy at a transcriptional level. FOXO3 activates gene expression of several autophagy genes, including ATG4, BECN1, and ULK1. mTOR inhibitors, such as rapamycin and Torin1 induce autophagy [57, 58]. Our validation of Torin1 treatment effects in neurons also found increased protein level of LC3, a central protein in the autophagy pathway. Moreover, we found genes involved in autophagy were co-enriched with those related to the mTOR pathway in the down-regulated modules from human RNA-Seq data (PFC_M23), as well as from theTorin1 treated neurons (Torin1_M6). Thus RNA levels of a subset of genes related to autophagy are likely to be downregulated by inhibition of the mTOR pathway. However, this is somewhat inconsistent with previous studies [57, 58] that showed opposite results and indicates that the regulation of autophagy by the mTOR pathway at the transcriptional level may need further investigation.

Many of the individuals with BPD in this study were treated with mood stabilizers and/or antidepressants. Rodrigo Machado-Vieira et al. [16] found the expression of AKT and mTOR mRNA in peripheral blood decreased during a depressive episode in patients taking the mood stabilizer lithium as compared to healthy controls. Kara et al. [59] confirmed that when the mTOR inhibitor, rapamycin and autophagy enhancers, temosirolimus were administered to animal models of mania and depression, rapamycin improved manic behaviour and termsirolimus improved depressive behaviour. Mood stabilizers and antidepressant are known to activate the mTOR pathway by inhibiting GSK-3β [60]. High-throughput data from animal studies also show that medications affect gene expression levels, particularly of genes related to mTOR pathway [61, 62]. Lithium induces differential expression of genes related to the mTOR and the Wnt pathways in the corpus callosum of rats [61] and the antidepressant, amitriptyline induces differential expression of genes related to the dopamine signaling cascade and ion channels in mice [62]. However, we found no significant correlation between mood stabilizers or antidepressants and the co-expression modules associated with BPD, indicating that the medications are unlikely to contribute to the differential expression of the modules. This is presumably because of the efficient adjustment of confounding effects on the RNA-Seq data by the SVA methods [20].

While our study presents novel and interesting findings there may be several limitations to the study. We did replicate two of the co-expression modules in the independent cohort of brain samples but we did not replicate the upregulated PFC_M5 module. Moreover, we did not identify a main regulator transcription factor for the module genes of the neuron-related PFC_M23 module. However, a previous report found a neural transcription factor, POU3F2, as a main regulator of a co-expression network related to glial and neural cell genesis in the brain of BPD [7]. Our study utilized the binding information of transcription factors, whereas the previous study integrally used binding information as well as the causal relationship between transcription factors and miRNA [7]. This may result in the different findings.

We used surrogate variables [20] as covariates to adjust for potential confounding effects on the RNA-seq data and the statistical method can efficiently remove potential confounding factors regardless of a priori information on the variables. However, abnormal expression of genes involved in metabolism as well as metabolic disturbances have been reported in BPD [63], and may contribute to differences in descriptive variables such as brain pH between BPD and unaffected controls [64]. Thus, adjustment of such variables by SVA may dilute the disease effect on gene expression profiling or on gene co-expression network analysis.

Our results indicated that genes involved similar mTOR and interrelated pathways were up-regulated in the endothelial cells and down-regulated in neurons in BPD. However, since we analysed RNA-seq data from bulk brain tissue samples that contain a heterogenous mixture of brain cell types, some of the genes involved in the same pathways may not be detected as differentially expressed genes or may not be included in the differentially expressed mTOR-related modules. Such dilution effects may cause some inconsistency in our results. Future RNA sequencing of sorted cell populations or of single cells may be powerful tools to identify further evidence to support our findings. These technologies, particularly in human post-mortem brain tissue will be critical for identifying differentially expressed genes and/or co-expression modules if the expression of the genes involved in the same pathway are changed in opposite directions in different cell types. The technical barriers will be removed eventually by advancing the technologies and will be employed in future studies.

## Supplementary information

Supplementary Figures

Supplementary Tables

Supplementary Methods
