# Correction: Differential expression of gene co-expression networks related to the mTOR signaling pathway in bipolar disorder

**DOI:** 10.1038/s41398-022-01979-x

**Published:** 2022-05-31

**Authors:** Sung Woo Park, Mi Kyoung Seo, Maree J. Webster, Jung Goo Lee, Sanghyeon Kim

**Affiliations:** 1 Department of Convergence Biomedical Science, College of Medicine, Inje University, 75 Bokji-ro, Busnajin-gu, Busan, 47392 Republic of Korea; 2 Paik Institute for Clinical Research, Inje University, 75 Bokji-ro, Busnajin-gu, Busan, 47392 Republic of Korea; 3 Stanley Brain Research Laboratory, Stanley Medical Research Institute, 9800 Medical Center Drive, Rockville, MD 20850 USA; 4 Department of Psychiatry, College of Medicine, Haeundae Paik Hospital, Inje University, 875 Haeun-daero, Haeundae-gu, Busan, 47227 Republic of Korea

**Keywords:** Bipolar disorder, Molecular neuroscience

Correction to: *Translational Psychiatry* 10.1038/s41398-022-01944-8, published online 04 May 2022

The original version of this article unfortunately contained a mistake. The main corresponding author’s (Sanghyeon Kim; kims@stanleyresearch.org) email link was missing. We apologize for the mistake. The original article has been corrected.

