# Supplementary material for: Differential expression of gene co-expression networks related to the mTOR signaling pathway in bipolar disorder
Source: Transl Psychiatry. 2022 May 4;12:184. doi: 10.1038/s41398-022-01944-8 (PMC9067344; doi:10.1038/s41398-022-01944-8)
Supplement: Supplementary file 1 — Supplementary Figures [file 41398_2022_1944_MOESM1_ESM.docx]

**Supplementary Figures**

**
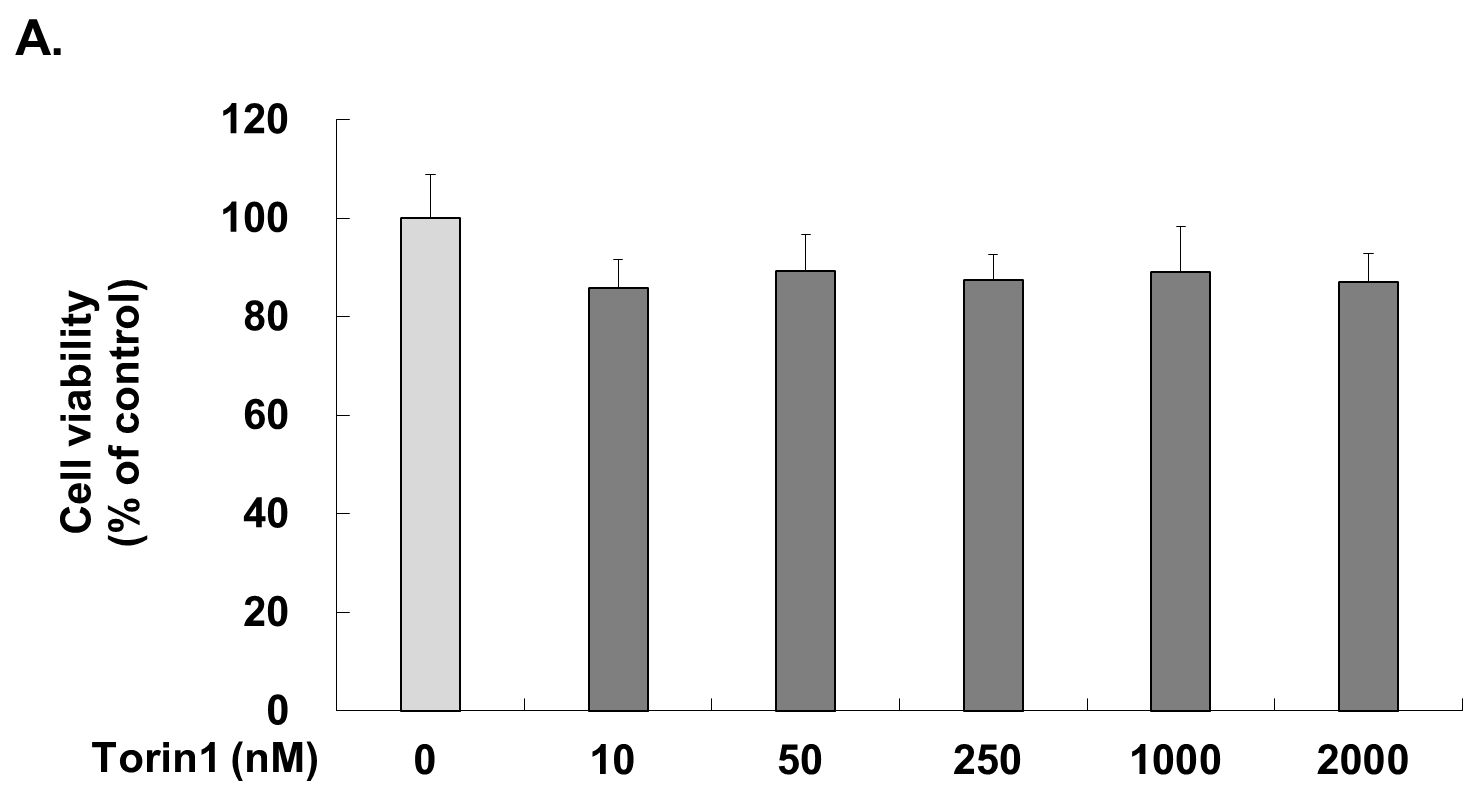
**


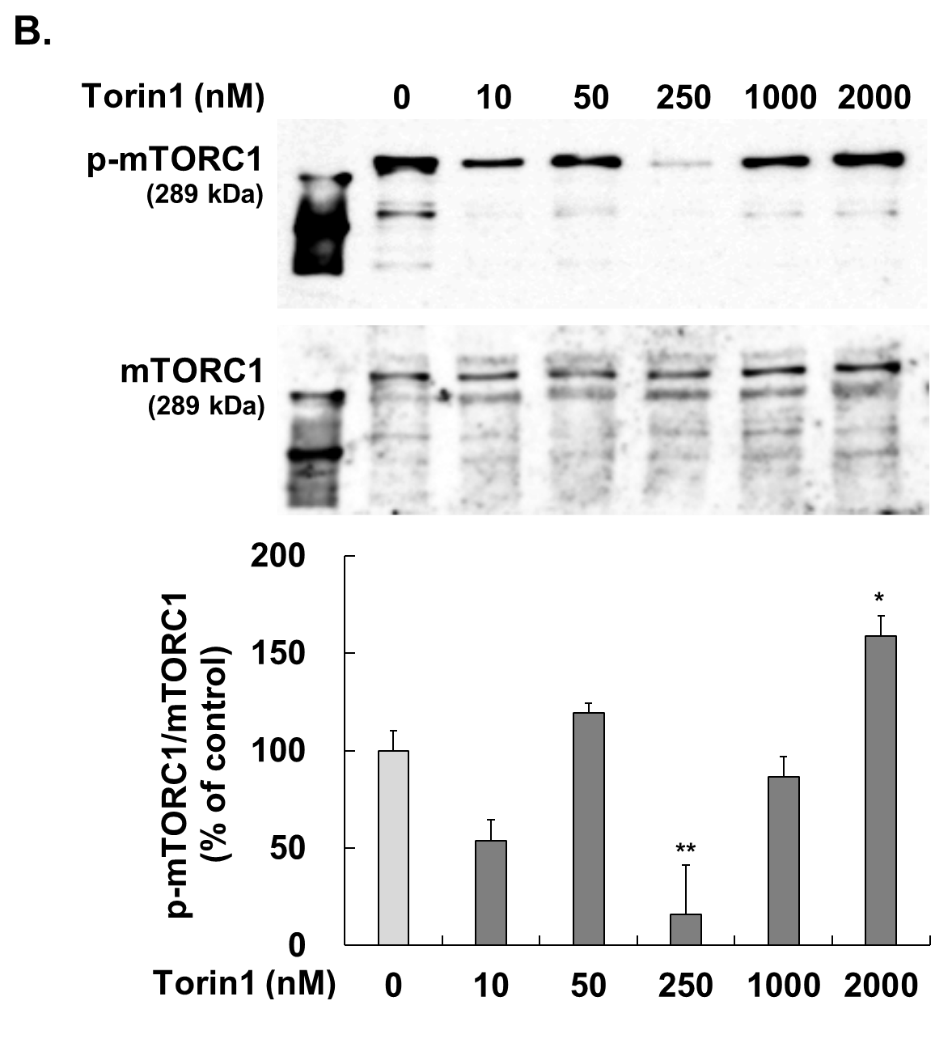


**Supplementary Figure 1**. Dose response of Torin1 to establish the optimal dose in primary neuronal cells.

Neuronal cells were treated with Torin1 (10, 50, 250, 1000 and 2000 nM) for 24 h. (A) To cytotoxicity, MTT assay was performed. Concentrations of Torin1 form 10 nM to 2 µM did not affect cell viability. (B) To measuring phospho-mTORC1 levels, cell lysates were SDS-PAGE and Western blot analyses. Torin1 of 250 nM decreased phospho-mTORC1 level. Based on these experiments, dose of 250 nM was chosen for further study. Results are presented as the mean ± SEM expressed as a percentage of the control cells (no drug) values. **p* < 0.05 or ***p* < 0.01 vs. control cells (One-way ANOVA followed by *post hoc* test).


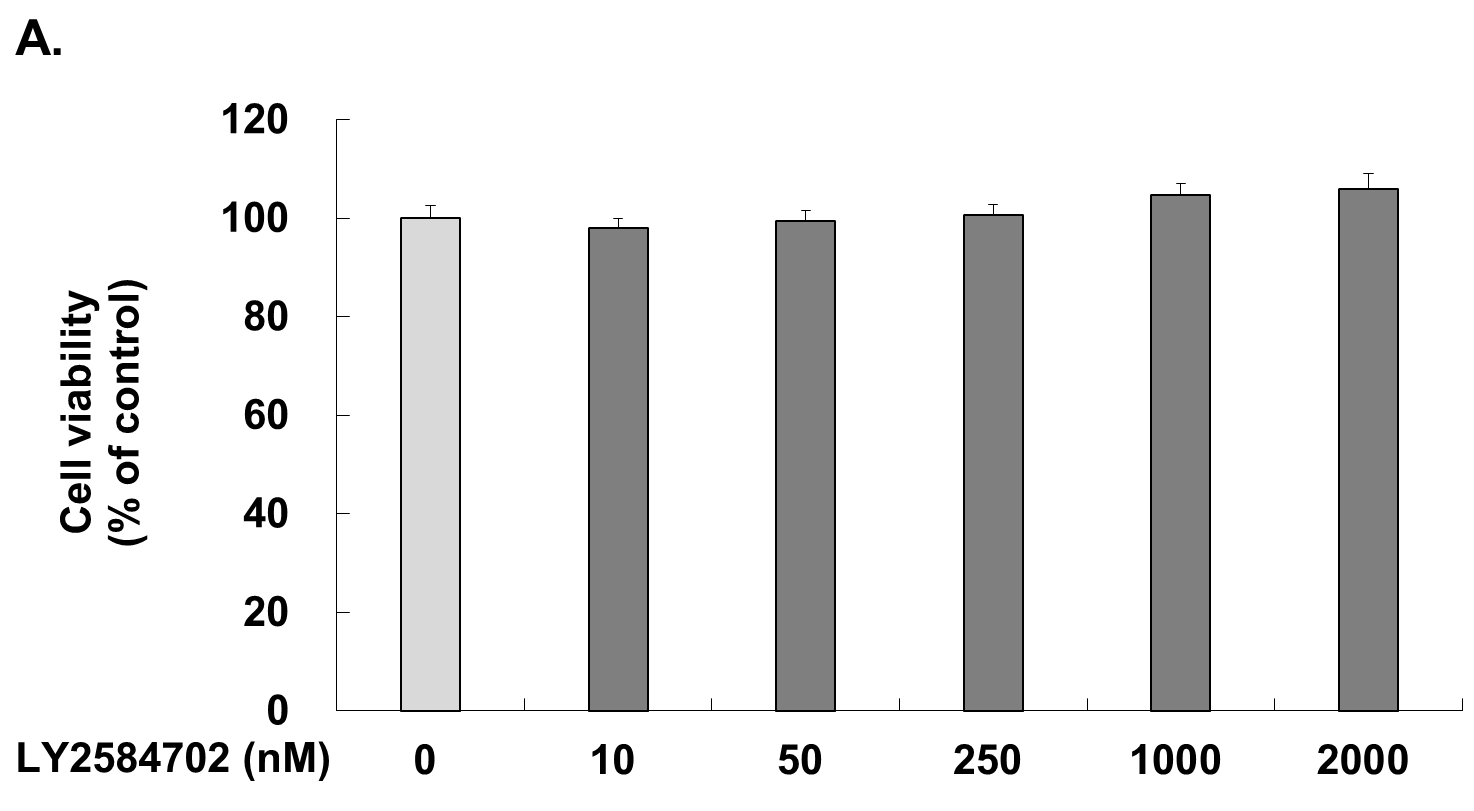


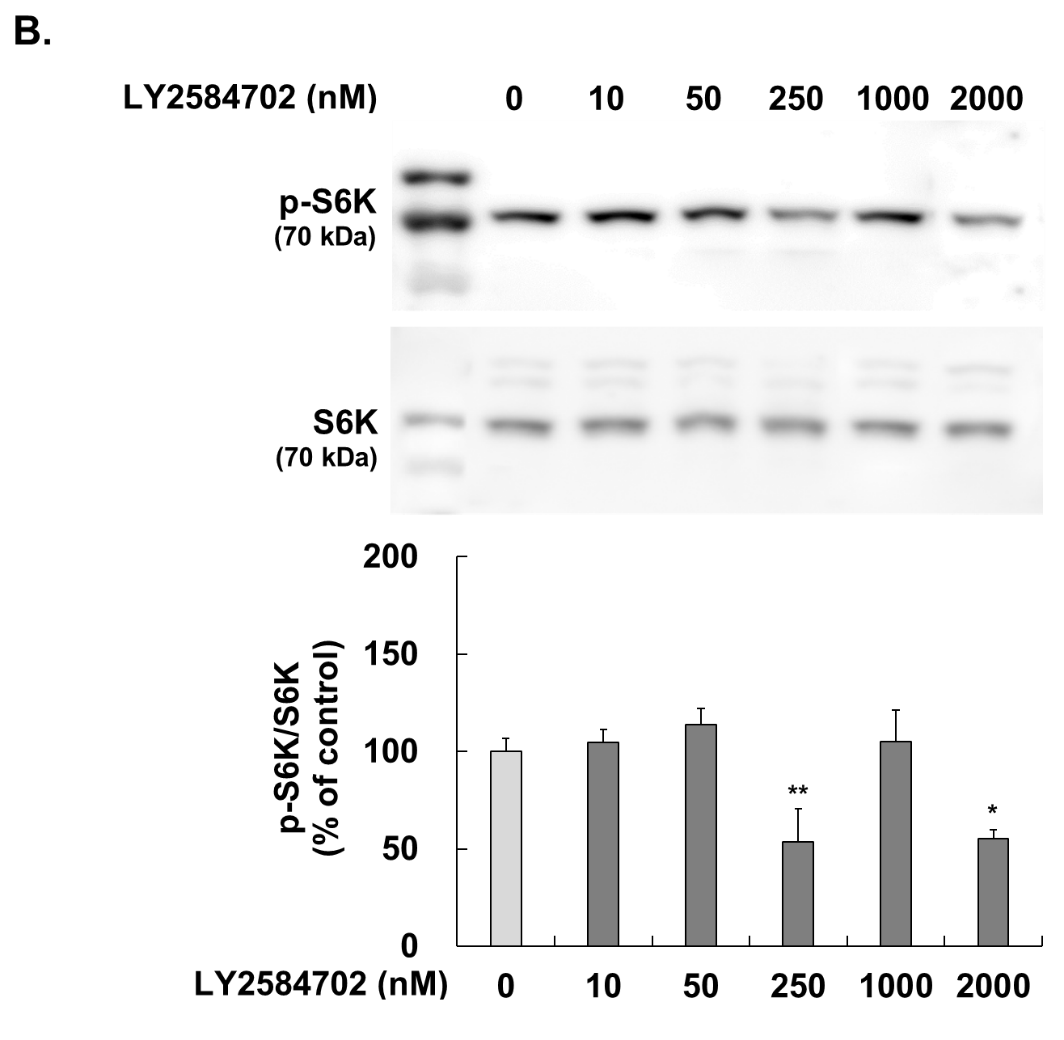


**Supplementary Figure 2.** Dose response of LY2584702 to establish the optimal dose in primary neuronal cells.

Neuronal cells were treated with LY2584702 (10, 50, 250, 1000 and 2000 nM) for 24 h. (A) To cytotoxicity, MTT assay was performed. Concentrations of LY2584702 form 10 nM to 2 µM did not affect cell viability. (B) To measuring phospho-S6K levels, cell lysates were SDS-PAGE and Western blot analyses. LY2584702 of 250 nM and 2 µM decreased phospho-S6K level. Based on these experiments, dose of 250 nM was chosen for further study. Results are presented as the mean ± SEM expressed as a percentage of the control cells (no drug) values. **p* < 0.05 vs. control cells (One-way ANOVA followed by *post hoc* test).

**
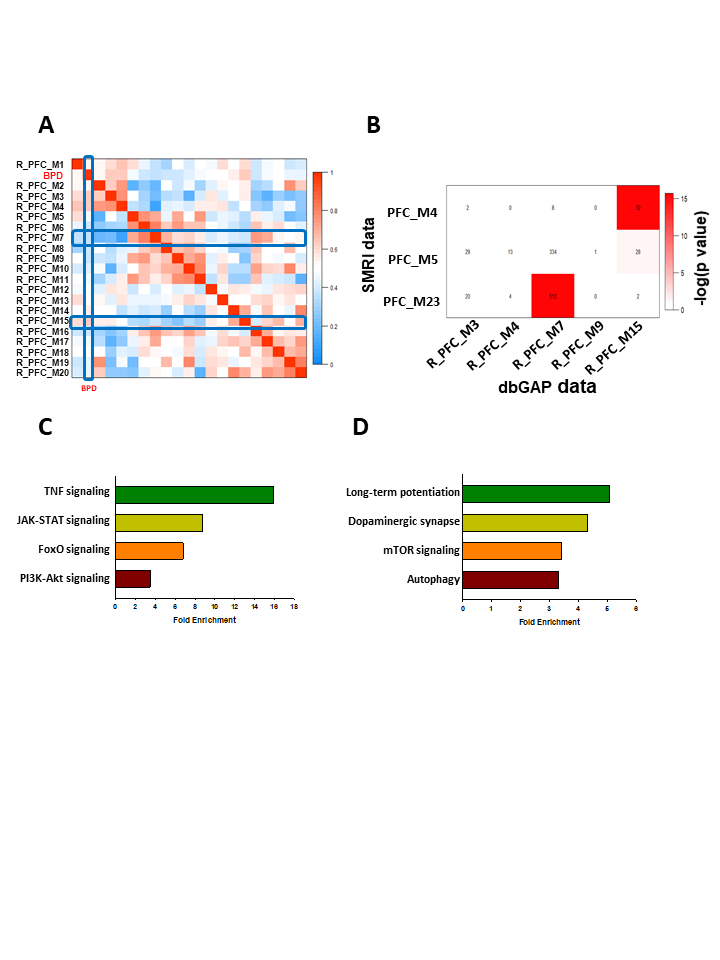
**

**Supplementary Figure 3**. **Replication of the mTOR pathway-related modules using an independent data set**

A, The eigengene adjacency heatmap of the modules significantly associated with BPD in the dbGAP RNA-seq data (accession number phs000979.v2.p2). B, Pairwise comparisons of the mTOR pathway-related modules associated with BPD in the SMRI RNA-seq data and the significant modules from the dbGAP RNA-seq data (accession number phs000979.v2.p2). The color code of the heatmap encodes −log (*P*-value). Fisher exact test was used to calculate the *P*-values for the overlap of the two modules. The numbers in the heatmap indicate gene counts in the intersection of two modules. Major KEGG pathways significantly enriched in the genes common to PFC_M4 module from SMRI data and R_PFC_M15 module dbGAP data (C) and PFC_M23 module from SMRI data and R_PFC_M7 module dbGAP data (D).

**A. Akt**


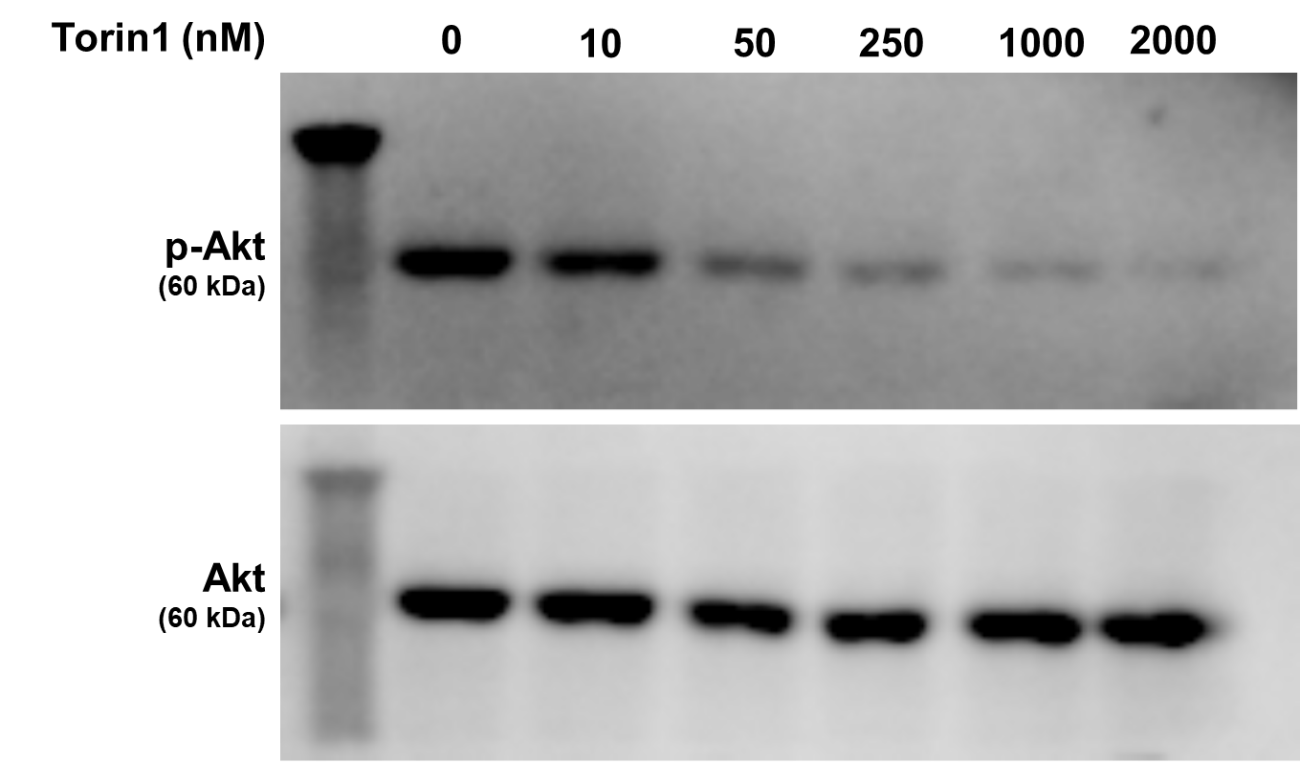


**B. mTORC1**


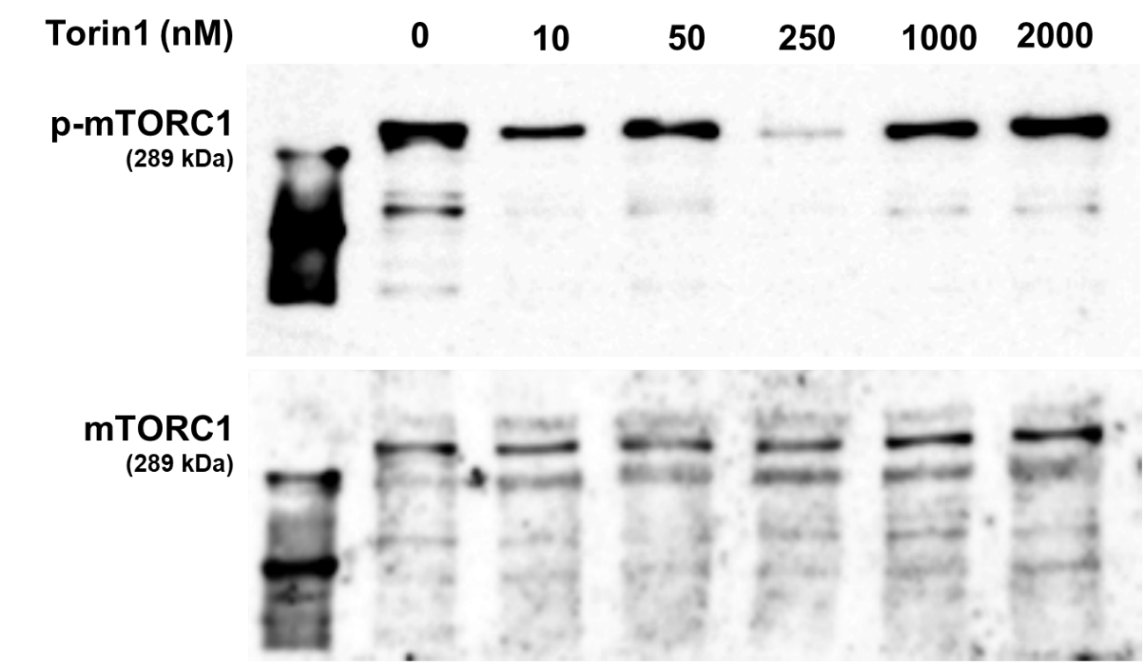


**C. S6K**


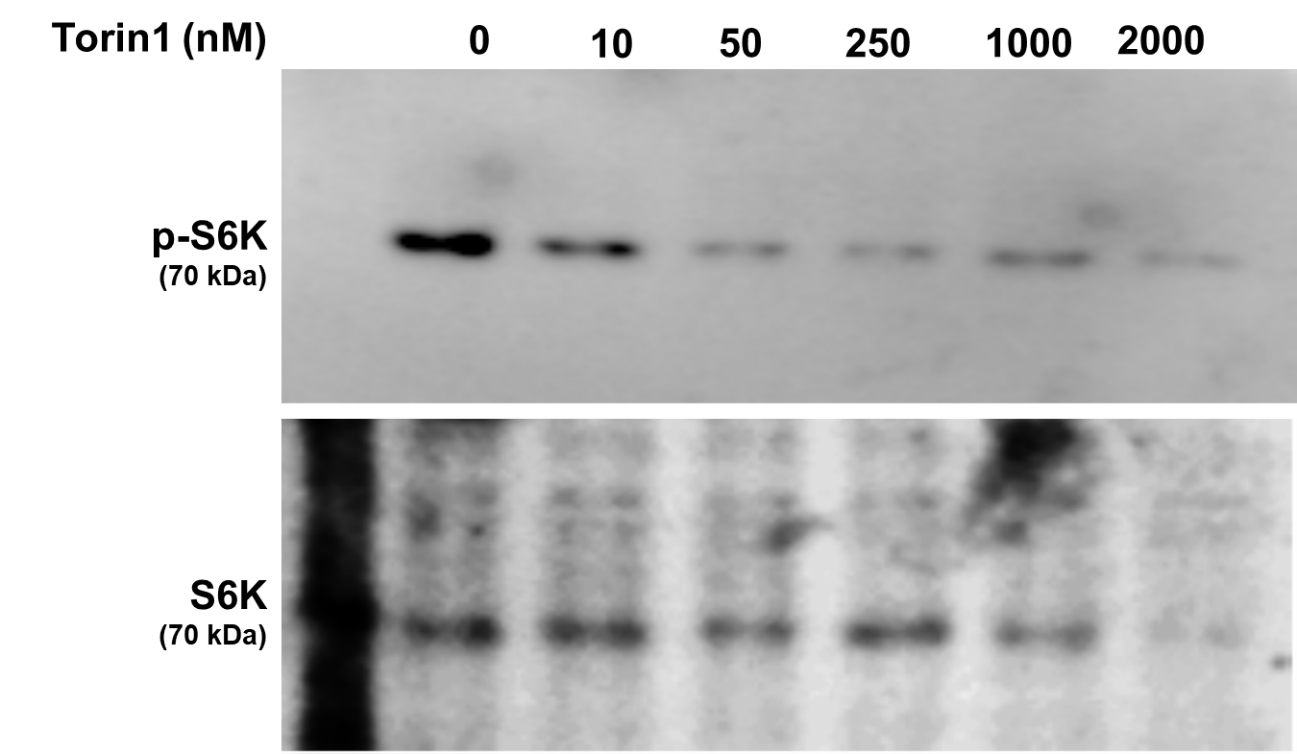


**D. PSD-95, LC3B, and Beclin1**


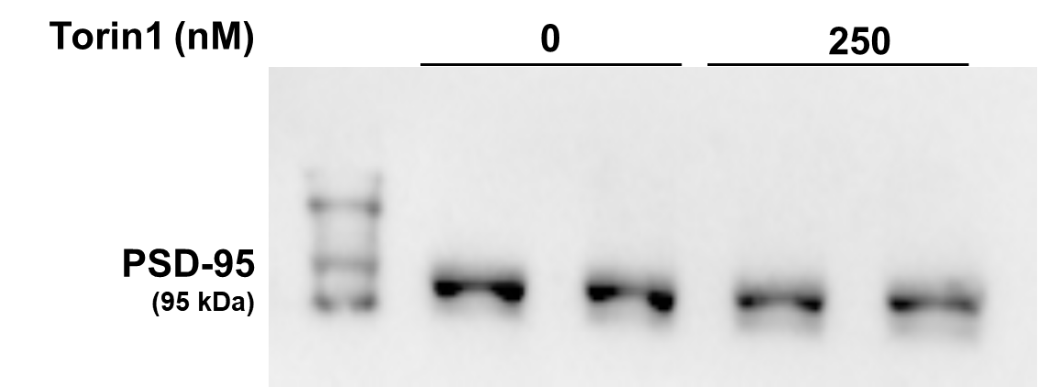


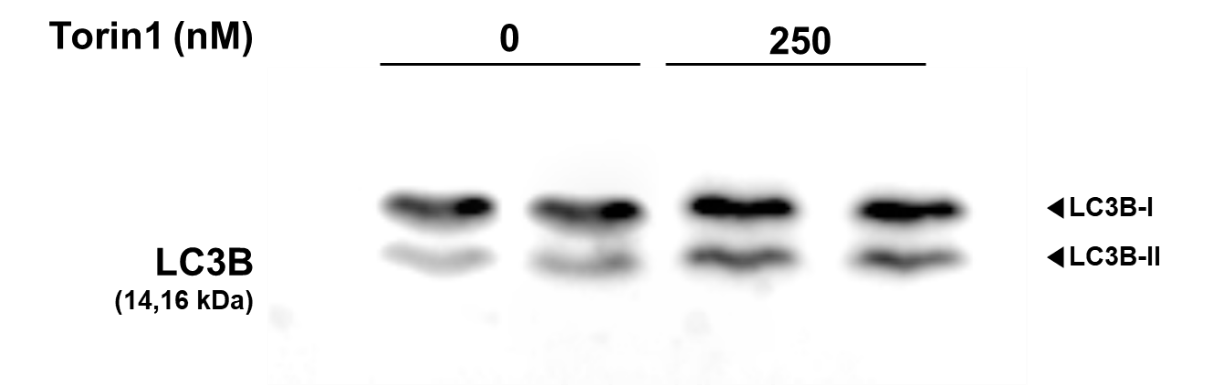


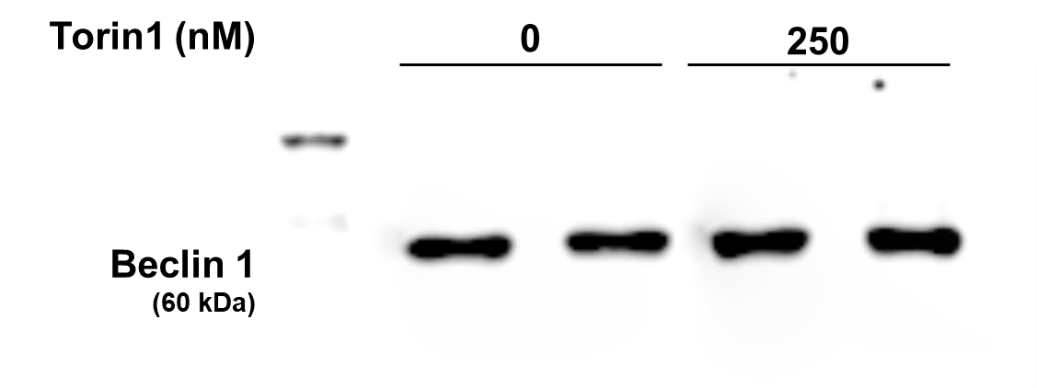


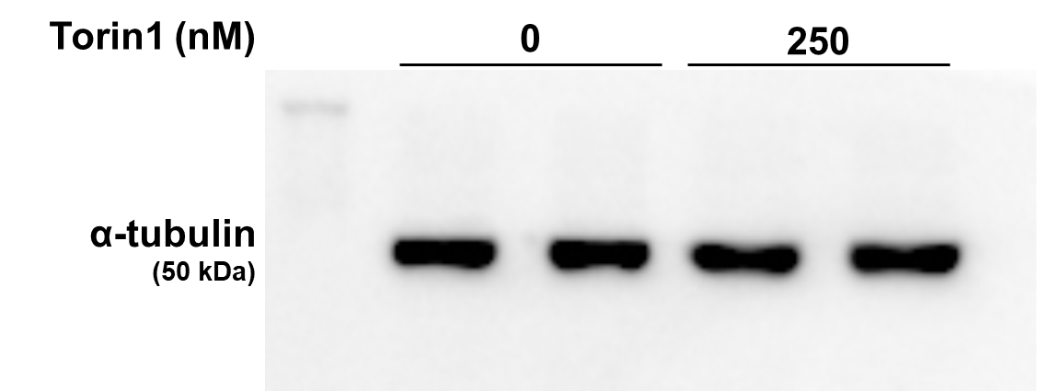


**Supplementary Figure 4.** The original images from which we constructed Figure 3A. Some wells were excluded because the corresponding experimental group (Torin 1 10, 50, 1000, and 2000 nM) are not treated.


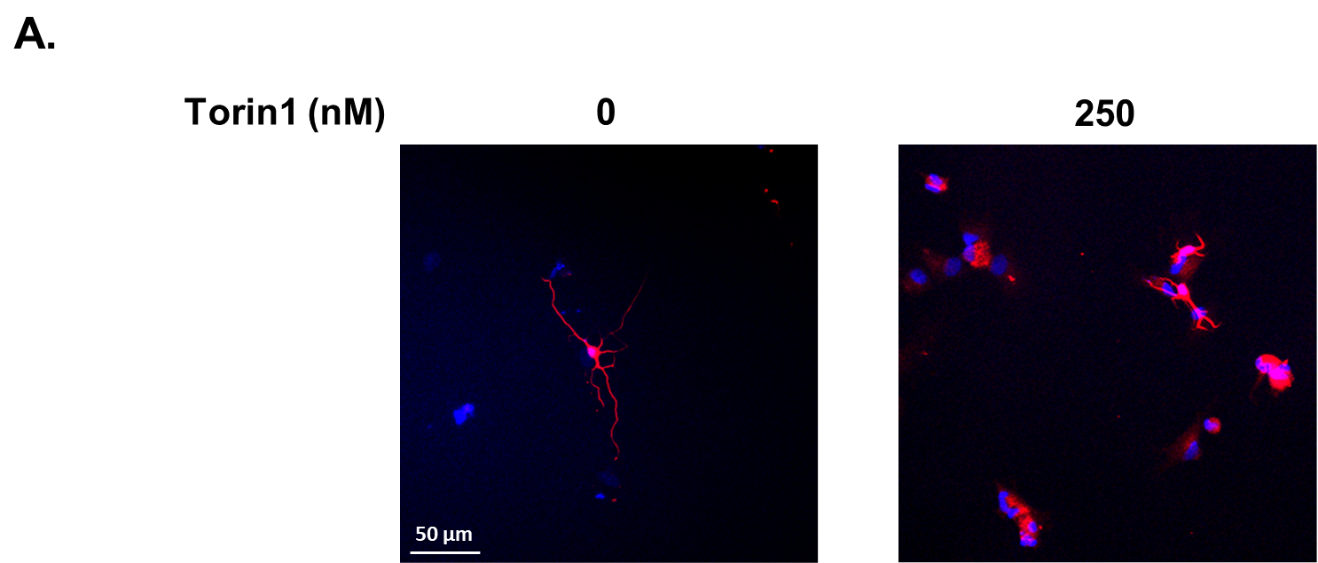


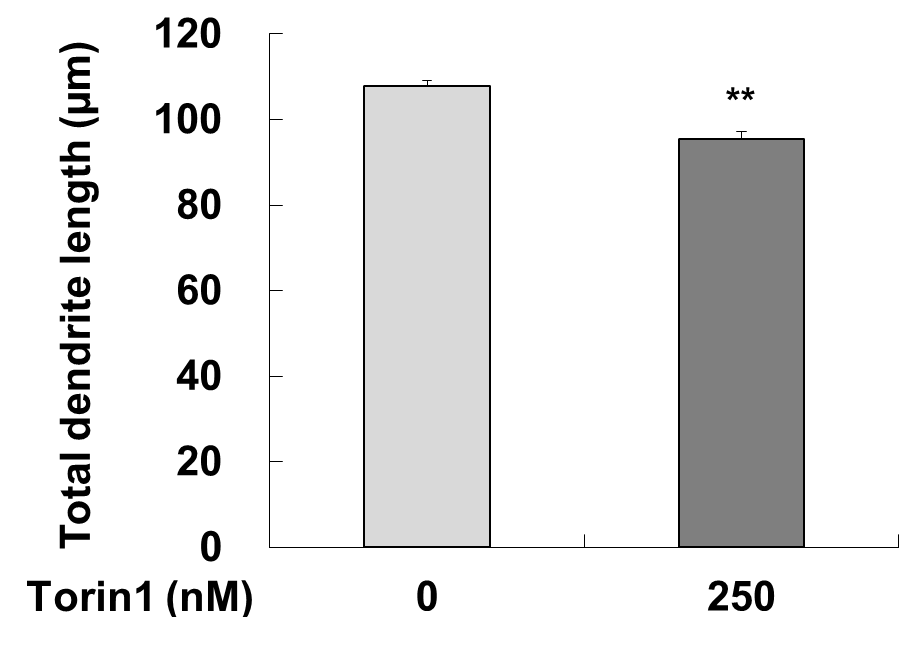


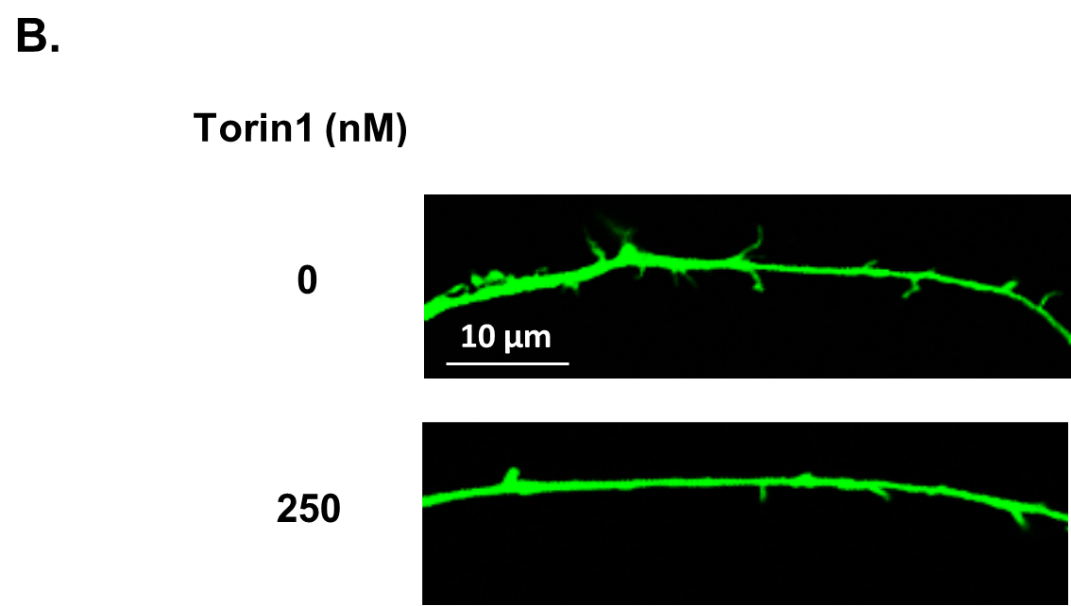


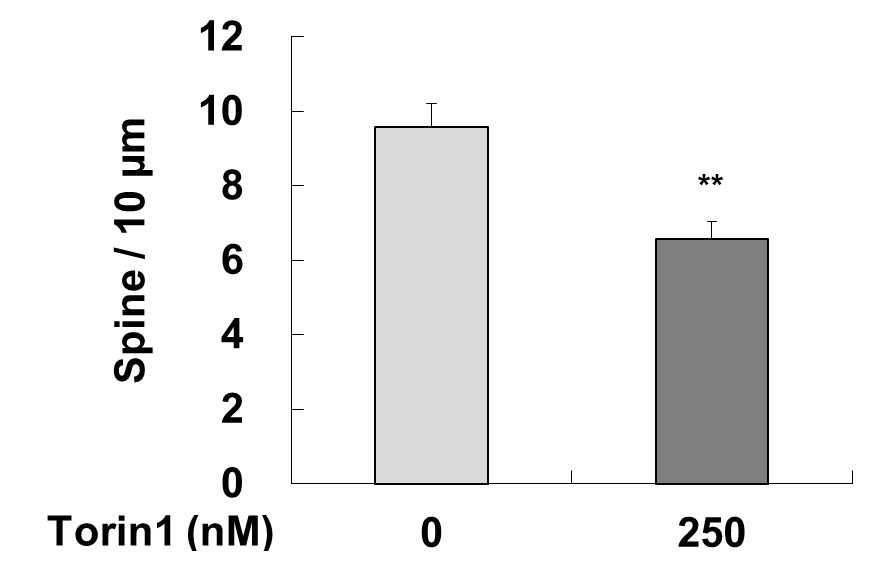


**Supplementary Figure 5.** Effects of Torin1 on total dendritic length and spine density in primary neuronal cells.

Neuronal cells were treated with Torin1 250 nM for 24 h. (A) In total, 100 cells of group were analyzed for total dendritic length. (B) In total, 20 dendritic segments per group were analyzed for spine density. Results are presented as the mean ± SEM. **p* < 0.05, ***p* < 0.01, *unpaired Student t test*.

**A. S6K**


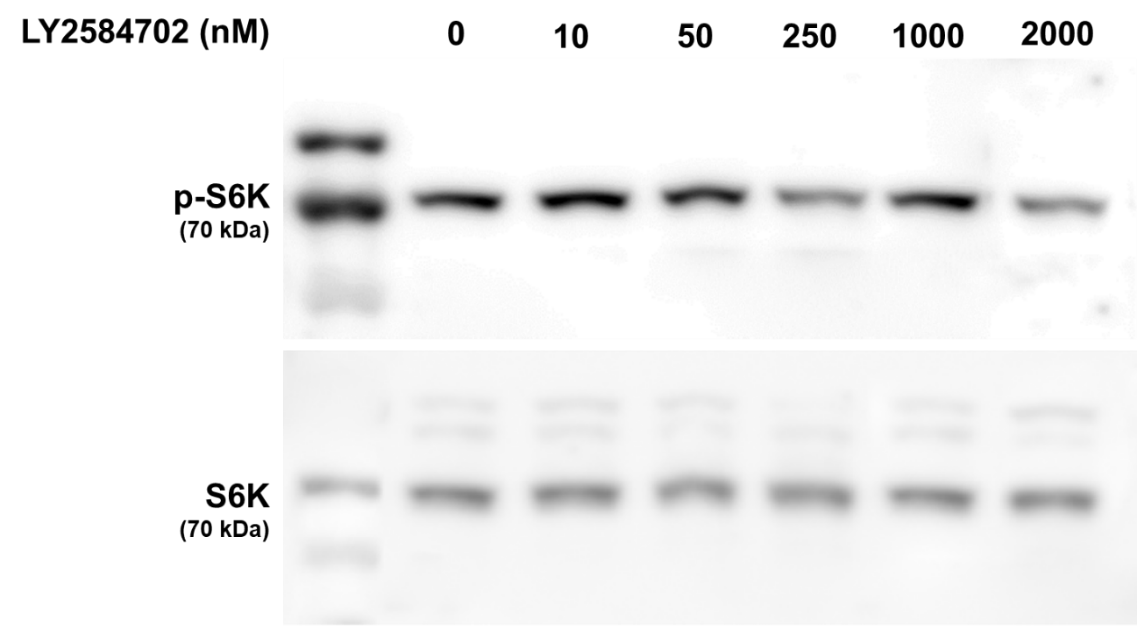


**B. S6**


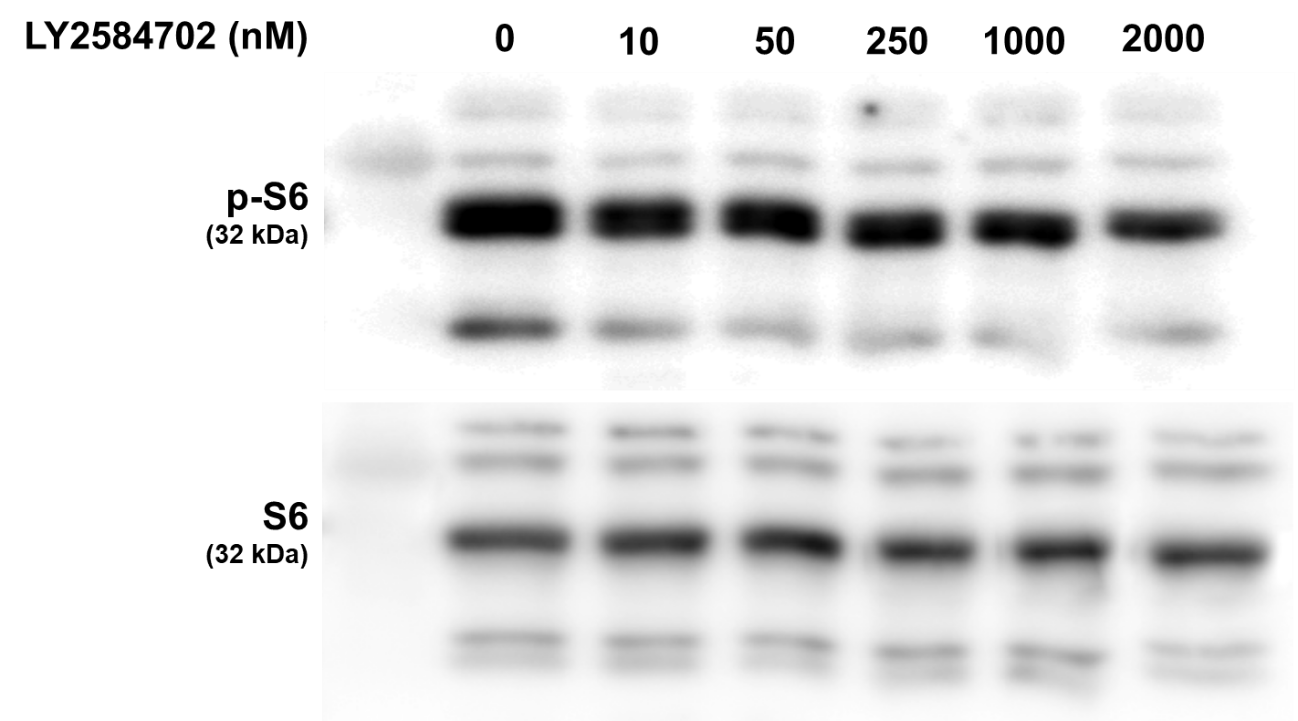


**C. GluA1 and PSD-95**


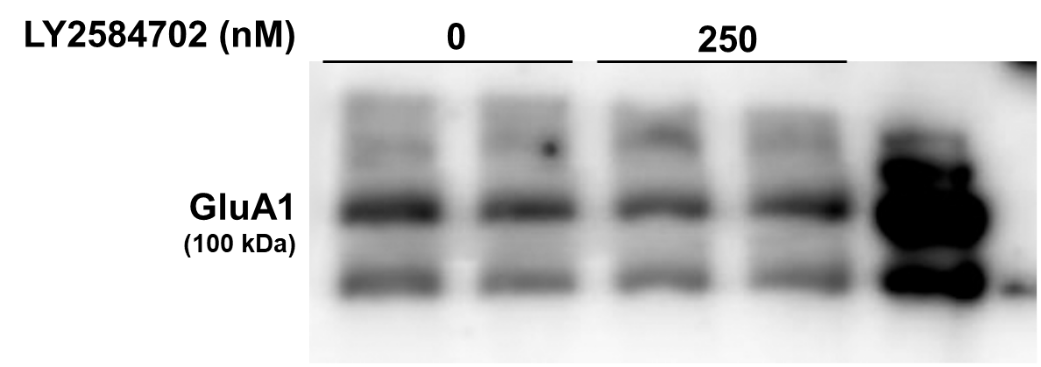


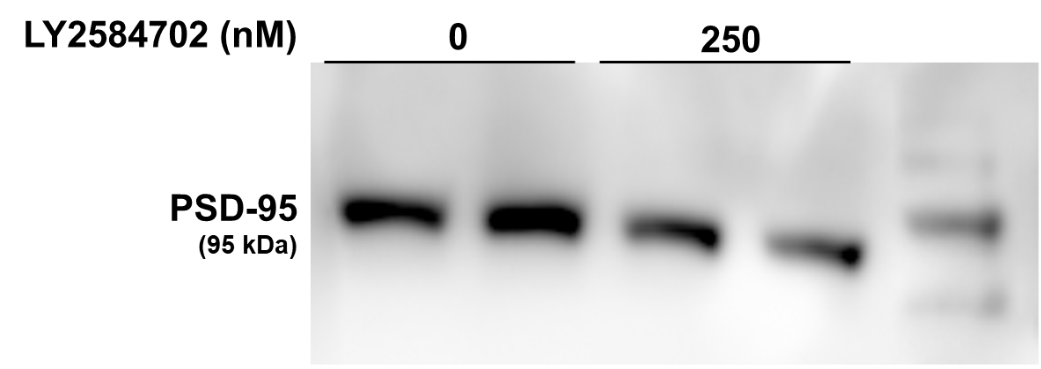


**
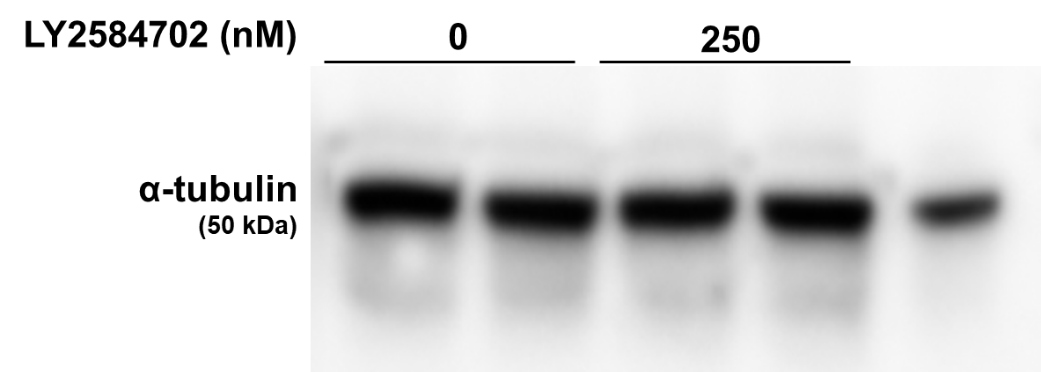
**

**Supplementary Figure 6.** The original images from which we constructed Figure 3B. Some wells were excluded because the corresponding experimental group (LY2584702 10, 50, 1000, and 2000 nM) are not treated.
