# Supplementary material for: Differential expression of gene co-expression networks related to the mTOR signaling pathway in bipolar disorder
Source: Transl Psychiatry. 2022 May 4;12:184. doi: 10.1038/s41398-022-01944-8 (PMC9067344; doi:10.1038/s41398-022-01944-8)
Supplement: Supplementary file 2 — Supplementary Tables [file 41398_2022_1944_MOESM2_ESM.docx]

**Supplementary Table 1**. Demographic and clinical variables for samples used in this study.

|  | Discovery study | | | Replication study | |
| --- | --- | --- | --- | --- | --- |
| Variables | BPD (SMRI) | NC (SMRI) | NC (Public) | BPD (dbGAP) | NC (dbGAP) |
|  | N=60 | N=66 | N=28 | N=55 | N=122 |
| Age | 44.4±11.8 | 47.3±8.9 | 57.4±9.5 | 39.8±11.8 | 42.6±11.8 |
| Sex (M/F) | 31/29 | 52/14 | 28/0 | 40/15 | 91/31 |
| Brain pH | 6.4±0.3 | 6.5±0.3 | N.A | 6.4±0.2 | 6.5±0.3 |
| PMI | 35.3±16.7 | 28.5± 14.4 | N.A | 28.1±11.6 | 29.4± 11.9 |
| Antipsychotic | 11021.2±21737.4 | N.A | N.A | N.A | N.A |
| Li (yes/no) | 16/44 | N.A | N.A | N.A | N.A |
| MS (yes/no) | 42/18 | N.A | N.A | N.A | N.A |
| AD (yes/no) | 34/26 | N.A | N.A | N.A | N.A |
| RIN | 8.1± 0.9 | 8.3± 0.7 | 7.8± 0.6 | N.A | N.A |

Values are mean ± S.D. BPD, bipolar disorder; NC, normal control; PMI, post-mortem interval; Antipsychotic, normalized as fluphenazine gram equivalent ; Li, lithium treatment; MS, mood stabilizer treatment; AD, antidepressant treatment; RIN, RNA integrity number

**Supplementary Table 2.**  KEGG pathways enriched in up-regulated genes in PFC of BPD as compared to controls.

| **Term_name** | **Term_id** | **Count** | **Fold Enrichment** | **Adjusted_p_value** |
| --- | --- | --- | --- | --- |
| Ribosome | KEGG:03010 | 67 | 3.4 | 4.4E-19 |
| Thermogenesis | KEGG:04714 | 50 | 1.7 | 0.02 |
| Insulin resistance | KEGG:04931 | 28 | 2.0 | 0.02 |

**Supplementary Table 3.**  KEGG pathways enriched in down-regulated genes in PFC of BPD as compared to controls.

| **Term_name** | **Term_id** | **Count** | **Fold Enrichment** | **Adjusted p_value** |
| --- | --- | --- | --- | --- |
| GABAergic synapse | KEGG:04727 | 19 | 3.2 | 0.002 |
| Spinocerebellar ataxia | KEGG:05017 | 23 | 2.4 | 0.006 |
| Ubiquitin mediated proteolysis | KEGG:04120 | 23 | 2.5 | 0.006 |
| Glutamatergic synapse | KEGG:04724 | 19 | 2.5 | 0.014 |
| Autophagy - animal | KEGG:04140 | 21 | 2.3 | 0.016 |
| Endocytosis | KEGG:04144 | 31 | 1.9 | 0.023 |
| Mitophagy - animal | KEGG:04137 | 13 | 2.9 | 0.023 |
| Shigellosis | KEGG:05131 | 30 | 1.9 | 0.028 |
| Salmonella infection | KEGG:05132 | 27 | 1.9 | 0.033 |
| Thyroid hormone signaling pathway | KEGG:04919 | 18 | 2.2 | 0.034 |
| Hedgehog signaling pathway | KEGG:04340 | 10 | 3.0 | 0.042 |
| Calcium signaling pathway | KEGG:04020 | 25 | 1.9 | 0.044 |
| Synaptic vesicle cycle | KEGG:04721 | 13 | 2.5 | 0.044 |
| Adrenergic signaling in cardiomyocytes | KEGG:04261 | 20 | 2.0 | 0.047 |
| Endocrine and other factor-regulated calcium reabsorption | KEGG:04961 | 10 | 2.8 | 0.049 |

**Supplementary Table 4.** Differentially expressed genes involved in the mTOR pathway

| Gene symbol | Gene name | logFC | FDR |
| --- | --- | --- | --- |
| CAB39L | protein kinase AMP-activated catalytic subunit alpha 2 | -0.16 | 1.69E-04 |
| PIK3CB | protein kinase C beta | -0.12 | 4.33E-05 |
| STK11 | calcium binding protein 39 like | -0.11 | 1.35E-02 |
| RPS6KA3 | 3-phosphoinositide dependent protein kinase 1 | -0.09 | 1.74E-03 |
| RHEB | ribosomal protein S6 | -0.09 | 2.56E-03 |
| ULK3 | phosphatidylinositol-4,5-bisphosphate 3-kinase catalytic subunit beta | -0.09 | 8.35E-03 |
| PRKCB | phosphoinositide-3-kinase regulatory subunit 1 | -0.08 | 7.43E-03 |
| PDPK1 | ribosomal protein S6 kinase A3 | -0.07 | 6.94E-03 |
| PIK3R1 | serine/threonine kinase 11 | -0.07 | 9.51E-03 |
| EIF4E | Ras related GTP binding A | -0.06 | 2.67E-02 |
| RRAGA | Ras homolog enriched in brain | -0.05 | 1.84E-02 |
| AKT3 | AKT serine/threonine kinase 3 | -0.05 | 2.65E-02 |
| ATP6V1D | ATPase H+ transporting V1 subunit D(ATP6V1D) | -0.05 | 4.67E-02 |
| ATP6V1A | ATPase H+ transporting V1 subunit A(ATP6V1A) | -0.09 | 3.72E-03 |
| ATP6V1B2 | ATPase H+ transporting V1 subunit B2(ATP6V1B2) | -0.07 | 4.14E-03 |
| PRKAA2 | unc-51 like kinase 3 | 0.1 | 3.77E-03 |
| RPS6 | eukaryotic translation initiation factor 4E binding protein 1 | 0.11 | 1.17E-02 |
| EIF4EBP1 | eukaryotic translation initiation factor 4E | 0.17 | 2.40E-02 |

logFC, log2 fold change between the groups; FDR, false discovery rate

**Supplementary Table 5.** Correlation coefficients between co-expression modules in the PFC and BPD or descriptive variables

| **Module** | **BP** | **Age** | **Sex** | **PMI** | **Brain pH** | **AP** | **RIN** | **Li** | **MS** | **AD** |
| --- | --- | --- | --- | --- | --- | --- | --- | --- | --- | --- |
| PFC_M1 | 0.32 | -0.32 | N.S | N.S | N.S | N.S | N.S | N.S | N.S | N.S |
| PFC_M2 | 0.24 | N.S | N.S | N.S | N.S | N.S | N.S | N.S | N.S | N.S |
| PFC_M3 | 0.31 | N.S | N.S | N.S | N.S | N.S | N.S | N.S | N.S | N.S |
| PFC_M4 | 0.41 | N.S | N.S | N.S | N.S | N.S | N.S | N.S | N.S | N.S |
| PFC_M5 | 0.47 | N.S | N.S | N.S | N.S | N.S | N.S | N.S | N.S | N.S |
| PFC_M6 | 0.32 | N.S | N.S | N.S | N.S | N.S | N.S | N.S | N.S | N.S |
| PFC_M7 | 0.36 | N.S | N.S | N.S | N.S | N.S | N.S | N.S | N.S | N.S |
| PFC_M8 | 0.26 | N.S | N.S | N.S | N.S | N.S | N.S | N.S | N.S | N.S |
| PFC_M9 | 0.27 | N.S | N.S | N.S | N.S | N.S | N.S | N.S | N.S | N.S |
| PFC_M10 | -0.27 | N.S | N.S | -0.27 | N.S | N.S | N.S | N.S | N.S | N.S |
| PFC_M11 | N.S | N.S | N.S | N.S | N.S | N.S | N.S | N.S | N.S | N.S |
| PFC_M12 | -0.31 | N.S | N.S | N.S | N.S | N.S | N.S | N.S | N.S | N.S |
| PFC_M13 | -0.27 | N.S | N.S | N.S | N.S | N.S | N.S | N.S | N.S | N.S |
| PFC_M14 | -0.38 | N.S | N.S | N.S | N.S | N.S | N.S | N.S | N.S | N.S |
| PFC_M15 | N.S | N.S | N.S | N.S | N.S | N.S | N.S | N.S | N.S | N.S |
| PFC_M16 | -0.29 | N.S | N.S | N.S | N.S | N.S | N.S | N.S | N.S | N.S |
| PFC_M17 | -0.3 | N.S | N.S | N.S | N.S | N.S | N.S | N.S | N.S | N.S |
| PFC_M18 | N.S | N.S | N.S | N.S | N.S | N.S | N.S | N.S | N.S | N.S |
| PFC_M19 | N.S | N.S | N.S | 0.27 | N.S | N.S | -0.26 | N.S | N.S | N.S |
| PFC_M20 | N.S | N.S | N.S | N.S | N.S | N.S | N.S | N.S | N.S | N.S |
| PFC_M21 | -0.33 | N.S | -0.93 | N.S | N.S | N.S | N.S | N.S | N.S | N.S |
| PFC_M22 | N.S | N.S | N.S | N.S | N.S | N.S | N.S | N.S | N.S | N.S |
| PFC_M23 | -0.4 | N.S | -0.26 | N.S | N.S | N.S | N.S | N.S | N.S | N.S |
| PFC_M24 | N.S | N.S | N.S | N.S | N.S | N.S | N.S | N.S | N.S | N.S |
| PFC_M25 | N.S | N.S | N.S | N.S | N.S | N.S | N.S | N.S | N.S | N.S |
| PFC_M26 | N.S | 0.26 | N.S | N.S | N.S | N.S | N.S | N.S | N.S | N.S |
| PFC_M27 | N.S | 0.34 | N.S | N.S | N.S | N.S | -0.32 | N.S | N.S | N.S |
| PFC_M28 | N.S | N.S | N.S | N.S | N.S | N.S | -0.25 | N.S | N.S | N.S |

N.S, not significant; AP, normalized as fluphenazine gram equivalent; RIN, RNA integrity number; Li, lithium treatment; MS, mood stabilizer treatment; AD, antidepressant treatment

**Supplementary Table 6.**  Consensus genes between BPD-associated modules and the differentially expressed genes

| Module | Down regulated genes (n=1463) | Up regulated genes (n=2475) |
| --- | --- | --- |
| PFC_M1 | 0 (P=1) | 71 (P<2.2e-16) |
| PFC_M2 | 5 (P=1) | 72 (P=4.2e-06) |
| PFC_M3 | 34 (P=0.18) | 154 (P<2.2e-16) |
| PFC_M4 | 14 (P=0.6) | 102 (P<2.2e-16) |
| PFC_M5 | 48 (P=1) | 706 (P<2.2e-16) |
| PFC_M6 | 3 (P=1) | 166 (P=1.5e-08) |
| PFC_M7 | 26 (P=0.002) | 50 (P=2.1e-07) |
| PFC_M8 | 0 (P=1) | 30 (P=6.1e-07) |
| PFC_M9 | 0 (P=1) | 13 (P=0.005) |
| PFC_M10 | 104 (P=4.2e-014) | 32 (P=1) |
| PFC_M12 | 87 (P<2.2e-16) | 29 (P=1) |
| PFC_M13 | 23 (P=4.2e-05) | 1 (P=1) |
| PFC_M14 | 191 (P<2.2e-16) | 88 (P=0.97) |
| PFC_M16 | 30 (P=7.1e-10) | 3 (P=1) |
| PFC_M17 | 30 (P=2.0e-14) | 3 (P=1) |
| PFC_M21 | 4 (P=0.6) | 9 (P=0.2) |
| PFC_M23 | 310 (P<2.2e-16) | 36 (P=1) |

**Supplementary Table 7.**  KEGG pathways enriched in the genes in the co-expression modules associated with BPD in PFC

| **Module** | **Term_name** | **Term_id** | **Count** | **Fold Enrichment** | **Adjusted** |
| --- | --- | --- | --- | --- | --- |
|  |  |  |  |  | **p_value** |
| **PFC_M2** | Protein processing in endoplasmic reticulum | KEGG:04141 | 14 | 5.3 | 7.13E-05 |
|  | Spliceosome | KEGG:03040 | 9 | 4.2 | 2.80E-02 |
| **PFC_M4** | TNF signaling pathway | KEGG:04668 | 12 | 9.6 | 5.83E-07 |
|  | Kaposi sarcoma-associated herpesvirus infection | KEGG:05167 | 12 | 5.8 | 8.43E-05 |
|  | Hepatitis C | KEGG:05160 | 10 | 5.8 | 5.00E-04 |
|  | Epstein-Barr virus infection | KEGG:05169 | 11 | 5 | 5.00E-04 |
|  | Fluid shear stress and atherosclerosis | KEGG:05418 | 9 | 5.9 | 7.00E-04 |
|  | MicroRNAs in cancer | KEGG:05206 | 11 | 4.5 | 8.00E-04 |
|  | PI3K-Akt signaling pathway | KEGG:04151 | 14 | 3.6 | 8.00E-04 |
|  | JAK-STAT signaling pathway | KEGG:04630 | 9 | 5 | 1.40E-03 |
|  | Pathways in cancer | KEGG:05200 | 17 | 2.9 | 1.40E-03 |
|  | Small cell lung cancer | KEGG:05222 | 7 | 6.8 | 1.40E-03 |
|  | Bladder cancer | KEGG:05219 | 5 | 10.9 | 1.40E-03 |
|  | NF-kappa B signaling pathway | KEGG:04064 | 7 | 6.3 | 1.70E-03 |
|  | AGE-RAGE signaling pathway in diabetic complications | KEGG:04933 | 7 | 6.3 | 1.70E-03 |
|  | Transcriptional misregulation in cancer | KEGG:05202 | 9 | 4.4 | 2.60E-03 |
|  | HIF-1 signaling pathway | KEGG:04066 | 7 | 5.7 | 2.60E-03 |
|  | Proteoglycans in cancer | KEGG:05205 | 9 | 4 | 4.20E-03 |
|  | MAPK signaling pathway | KEGG:04010 | 11 | 3.3 | 4.20E-03 |
|  | Hepatitis B | KEGG:05161 | 8 | 4.4 | 4.20E-03 |
|  | Cytokine-cytokine receptor interaction | KEGG:04060 | 11 | 3.4 | 4.20E-03 |
|  | Influenza A | KEGG:05164 | 8 | 4.3 | 4.90E-03 |
|  | Salmonella infection | KEGG:05132 | 9 | 3.8 | 5.40E-03 |
|  | Human T-cell leukemia virus 1 infection | KEGG:05166 | 9 | 3.7 | 5.50E-03 |
|  | Thyroid cancer | KEGG:05216 | 4 | 9.7 | 5.90E-03 |
|  | Human cytomegalovirus infection | KEGG:05163 | 9 | 3.6 | 6.10E-03 |
|  | Non-small cell lung cancer | KEGG:05223 | 5 | 6.8 | 6.10E-03 |
|  | p53 signaling pathway | KEGG:04115 | 5 | 6.2 | 8.70E-03 |
|  | Pancreatic cancer | KEGG:05212 | 5 | 5.9 | 1.03E-02 |
|  | Chronic myeloid leukemia | KEGG:05220 | 5 | 5.9 | 1.03E-02 |
|  | Osteoclast differentiation | KEGG:04380 | 6 | 4.3 | 1.72E-02 |
|  | Rheumatoid arthritis | KEGG:05323 | 5 | 5.1 | 1.81E-02 |
|  | FoxO signaling pathway | KEGG:04068 | 6 | 4.2 | 1.81E-02 |
|  | Endometrial cancer | KEGG:05213 | 4 | 6.2 | 2.29E-02 |
|  | Apoptosis | KEGG:04210 | 6 | 3.9 | 2.30E-02 |
|  | Viral protein interaction with cytokine and cytokine receptor | KEGG:04061 | 5 | 4.6 | 2.58E-02 |
|  | Cell adhesion molecules (CAMs) | KEGG:04514 | 6 | 3.7 | 2.86E-02 |
|  | Th17 cell differentiation | KEGG:04659 | 5 | 4.3 | 3.13E-02 |
|  | Acute myeloid leukemia | KEGG:05221 | 4 | 5.3 | 3.31E-02 |
|  | Insulin resistance | KEGG:04931 | 5 | 4.1 | 3.38E-02 |
|  | Adipocytokine signaling pathway | KEGG:04920 | 4 | 5.3 | 3.38E-02 |
|  | Toxoplasmosis | KEGG:05145 | 5 | 4.1 | 3.43E-02 |
|  | Cellular senescence | KEGG:04218 | 6 | 3.4 | 3.80E-02 |
|  | Rap1 signaling pathway | KEGG:04015 | 7 | 3 | 3.96E-02 |
|  | Human papillomavirus infection | KEGG:05165 | 9 | 2.4 | 4.83E-02 |
| **PFC_M5** | Ribosome | KEGG:03010 | 87 | 9 | 4.53E-65 |
|  | Oxidative phosphorylation | KEGG:00190 | 45 | 4.7 | 2.17E-17 |
|  | Thermogenesis | KEGG:04714 | 58 | 3.5 | 7.62E-16 |
|  | Huntington disease | KEGG:05016 | 51 | 3.6 | 2.00E-14 |
|  | Parkinson disease | KEGG:05012 | 40 | 3.9 | 1.20E-12 |
|  | Alzheimer disease | KEGG:05010 | 40 | 3.2 | 7.09E-10 |
|  | Proteasome | KEGG:03050 | 17 | 5.2 | 2.24E-07 |
|  | Non-alcoholic fatty liver disease (NAFLD) | KEGG:04932 | 31 | 2.9 | 2.15E-06 |
|  | Spliceosome | KEGG:03040 | 27 | 2.8 | 2.72E-05 |
|  | Valine, leucine and isoleucine degradation | KEGG:00280 | 12 | 3.5 | 3.40E-03 |
|  | Fatty acid degradation | KEGG:00071 | 11 | 3.5 | 6.00E-03 |
|  | Metabolic pathways | KEGG:01100 | 137 | 1.3 | 1.80E-02 |
|  | Cardiac muscle contraction | KEGG:04260 | 15 | 2.4 | 2.65E-02 |
|  | beta-Alanine metabolism | KEGG:00410 | 8 | 3.6 | 2.72E-02 |
|  | Propanoate metabolism | KEGG:00640 | 8 | 3.3 | 4.83E-02 |
|  | Vibrio cholerae infection | KEGG:05110 | 10 | 2.8 | 4.99E-02 |
| **PFC_M7** | DNA replication | KEGG:03030 | 4 | 15.5 | 2.17E-02 |
| **PFC_M11** | Mineral absorption | KEGG:04978 | 4 | 9.9 | 4.81E-02 |
|  | Proximal tubule bicarbonate reclamation | KEGG:04964 | 3 | 18.8 | 4.81E-02 |
| **PFC_M14** | Metabolic pathways | KEGG:01100 | 79 | 1.5 | 9.50E-03 |
|  | Biosynthesis of amino acids | KEGG:01230 | 10 | 3.8 | 2.30E-02 |
|  | Carbon metabolism | KEGG:01200 | 13 | 3.2 | 2.30E-02 |
| **PFC_M16** | Vascular smooth muscle contraction | KEGG:04270 | 6 | 7.4 | 1.98E-02 |
| **PFC_M17** | Neuroactive ligand-receptor interaction | KEGG:04080 | 11 | 9.7 | 1.88E-07 |
|  | GABAergic synapse | KEGG:04727 | 4 | 13.5 | 4.90E-03 |
|  | Taurine and hypotaurine metabolism | KEGG:00430 | 2 | 59.9 | 7.90E-03 |
|  | Butanoate metabolism | KEGG:00650 | 2 | 21.4 | 4.70E-02 |
|  | beta-Alanine metabolism | KEGG:00410 | 2 | 19.3 | 4.70E-02 |
| **PFC_M23** | Long-term potentiation | KEGG:04720 | 18 | 6.4 | 3.95E-08 |
|  | Calcium signaling pathway | KEGG:04020 | 27 | 3.4 | 3.29E-06 |
|  | Adrenergic signaling in cardiomyocytes | KEGG:04261 | 23 | 3.7 | 4.35E-06 |
|  | Synaptic vesicle cycle | KEGG:04721 | 16 | 4.9 | 5.01E-06 |
|  | Dopaminergic synapse | KEGG:04728 | 21 | 3.8 | 5.01E-06 |
|  | Oxytocin signaling pathway | KEGG:04921 | 23 | 3.6 | 5.01E-06 |
|  | cGMP-PKG signaling pathway | KEGG:04022 | 23 | 3.3 | 1.27E-05 |
|  | GnRH signaling pathway | KEGG:04912 | 16 | 4.1 | 3.47E-05 |
|  | Endocrine and other factor-regulated calcium reabsorption | KEGG:04961 | 12 | 5.4 | 3.47E-05 |
|  | cAMP signaling pathway | KEGG:04024 | 26 | 2.9 | 3.47E-05 |
|  | Oocyte meiosis | KEGG:04114 | 19 | 3.6 | 3.47E-05 |
|  | Circadian entrainment | KEGG:04713 | 16 | 4 | 5.24E-05 |
|  | Aldosterone synthesis and secretion | KEGG:04925 | 16 | 3.9 | 5.57E-05 |
|  | Salivary secretion | KEGG:04970 | 15 | 4 | 6.63E-05 |
|  | Inflammatory mediator regulation of TRP channels | KEGG:04750 | 16 | 3.8 | 6.63E-05 |
|  | Amphetamine addiction | KEGG:05031 | 13 | 4.5 | 7.85E-05 |
|  | Gastric acid secretion | KEGG:04971 | 13 | 4.2 | 2.00E-04 |
|  | Spinocerebellar ataxia | KEGG:05017 | 15 | 3.7 | 2.00E-04 |
|  | Insulin secretion | KEGG:04911 | 14 | 3.9 | 2.00E-04 |
|  | Apelin signaling pathway | KEGG:04371 | 18 | 3.2 | 2.00E-04 |
|  | GABAergic synapse | KEGG:04727 | 14 | 3.8 | 2.00E-04 |
|  | Pancreatic secretion | KEGG:04972 | 15 | 3.5 | 2.00E-04 |
|  | Glutamatergic synapse | KEGG:04724 | 16 | 3.4 | 2.00E-04 |
|  | mTOR signaling pathway | KEGG:04150 | 19 | 3 | 2.00E-04 |
|  | Axon guidance | KEGG:04360 | 21 | 2.8 | 2.00E-04 |
|  | Long-term depression | KEGG:04730 | 11 | 4.5 | 3.00E-04 |
|  | ErbB signaling pathway | KEGG:04012 | 13 | 3.7 | 4.00E-04 |
|  | Autophagy - animal | KEGG:04140 | 17 | 3 | 5.00E-04 |
|  | Phosphatidylinositol signaling system | KEGG:04070 | 14 | 3.4 | 6.00E-04 |
|  | Cholinergic synapse | KEGG:04725 | 15 | 3.2 | 6.00E-04 |
|  | Gap junction | KEGG:04540 | 13 | 3.5 | 6.00E-04 |
|  | Melanogenesis | KEGG:04916 | 14 | 3.3 | 6.00E-04 |
|  | Glucagon signaling pathway | KEGG:04922 | 14 | 3.2 | 1.00E-03 |
|  | Renin secretion | KEGG:04924 | 11 | 3.8 | 1.00E-03 |
|  | Insulin signaling pathway | KEGG:04910 | 16 | 2.8 | 1.30E-03 |
|  | Nicotine addiction | KEGG:05033 | 8 | 4.8 | 1.60E-03 |
|  | Serotonergic synapse | KEGG:04726 | 14 | 3 | 1.90E-03 |
|  | Proteoglycans in cancer | KEGG:05205 | 20 | 2.4 | 2.20E-03 |
|  | T cell receptor signaling pathway | KEGG:04660 | 13 | 3 | 2.50E-03 |
|  | Wnt signaling pathway | KEGG:04310 | 17 | 2.5 | 2.60E-03 |
|  | C-type lectin receptor signaling pathway | KEGG:04625 | 13 | 3 | 2.60E-03 |
|  | MAPK signaling pathway | KEGG:04010 | 25 | 2 | 3.90E-03 |
|  | Epithelial cell signaling in Helicobacter pylori infection | KEGG:05120 | 10 | 3.4 | 4.10E-03 |
|  | VEGF signaling pathway | KEGG:04370 | 9 | 3.7 | 4.60E-03 |
|  | Progesterone-mediated oocyte maturation | KEGG:04914 | 12 | 2.9 | 4.80E-03 |
|  | Cellular senescence | KEGG:04218 | 16 | 2.4 | 5.60E-03 |
|  | Glioma | KEGG:05214 | 10 | 3.2 | 6.50E-03 |
|  | Vascular smooth muscle contraction | KEGG:04270 | 14 | 2.5 | 7.10E-03 |
|  | Growth hormone synthesis, secretion and action | KEGG:04935 | 13 | 2.6 | 7.30E-03 |
|  | GnRH secretion | KEGG:04929 | 9 | 3.4 | 7.30E-03 |
|  | Neurotrophin signaling pathway | KEGG:04722 | 13 | 2.6 | 7.60E-03 |
|  | Human T-cell leukemia virus 1 infection | KEGG:05166 | 19 | 2.1 | 9.40E-03 |
|  | Dilated cardiomyopathy (DCM) | KEGG:05414 | 11 | 2.8 | 1.06E-02 |
|  | Alzheimer disease | KEGG:05010 | 16 | 2.2 | 1.10E-02 |
|  | Central carbon metabolism in cancer | KEGG:05230 | 9 | 3.1 | 1.14E-02 |
|  | Colorectal cancer | KEGG:05210 | 10 | 2.8 | 1.52E-02 |
|  | Cardiac muscle contraction | KEGG:04260 | 10 | 2.8 | 1.52E-02 |
|  | Carbohydrate digestion and absorption | KEGG:04973 | 7 | 3.6 | 1.56E-02 |
|  | Chagas disease (American trypanosomiasis) | KEGG:05142 | 11 | 2.6 | 1.56E-02 |
|  | Ras signaling pathway | KEGG:04014 | 19 | 2 | 1.71E-02 |
|  | Salmonella infection | KEGG:05132 | 18 | 2 | 1.71E-02 |
|  | Sphingolipid signaling pathway | KEGG:04071 | 12 | 2.4 | 1.80E-02 |
|  | Thyroid cancer | KEGG:05216 | 6 | 3.9 | 1.80E-02 |
|  | Thyroid hormone signaling pathway | KEGG:04919 | 12 | 2.4 | 1.80E-02 |
|  | Pancreatic cancer | KEGG:05212 | 9 | 2.8 | 1.88E-02 |
|  | Vibrio cholerae infection | KEGG:05110 | 7 | 3.4 | 1.96E-02 |
|  | Shigellosis | KEGG:05131 | 19 | 1.9 | 1.97E-02 |
|  | Estrogen signaling pathway | KEGG:04915 | 13 | 2.3 | 1.98E-02 |
|  | Collecting duct acid secretion | KEGG:04966 | 5 | 4.4 | 1.98E-02 |
|  | HIF-1 signaling pathway | KEGG:04066 | 11 | 2.4 | 2.36E-02 |
|  | Endocrine resistance | KEGG:01522 | 10 | 2.5 | 2.70E-02 |
|  | Human immunodeficiency virus 1 infection | KEGG:05170 | 17 | 1.9 | 2.72E-02 |
|  | Renal cell carcinoma | KEGG:05211 | 8 | 2.8 | 2.76E-02 |
|  | Synthesis and degradation of ketone bodies | KEGG:00072 | 3 | 7.2 | 2.76E-02 |
|  | Relaxin signaling pathway | KEGG:04926 | 12 | 2.2 | 2.76E-02 |
|  | AGE-RAGE signaling pathway in diabetic complications | KEGG:04933 | 10 | 2.4 | 3.34E-02 |
|  | Retrograde endocannabinoid signaling | KEGG:04723 | 13 | 2.1 | 3.34E-02 |
|  | Amyotrophic lateral sclerosis (ALS) | KEGG:05014 | 7 | 2.9 | 3.42E-02 |
|  | Longevity regulating pathway | KEGG:04211 | 9 | 2.5 | 3.74E-02 |
|  | Type II diabetes mellitus | KEGG:04930 | 6 | 3.2 | 3.82E-02 |
|  | Cushing syndrome | KEGG:04934 | 13 | 2 | 3.93E-02 |
|  | Inositol phosphate metabolism | KEGG:00562 | 8 | 2.6 | 4.11E-02 |
|  | Morphine addiction | KEGG:05032 | 9 | 2.4 | 4.11E-02 |
|  | Hypertrophic cardiomyopathy (HCM) | KEGG:05410 | 9 | 2.4 | 4.35E-02 |
|  | Parathyroid hormone synthesis, secretion and action | KEGG:04928 | 10 | 2.3 | 4.41E-02 |
|  | Arrhythmogenic right ventricular cardiomyopathy (ARVC) | KEGG:05412 | 8 | 2.5 | 4.85E-02 |
|  | Rap1 signaling pathway | KEGG:04015 | 16 | 1.8 | 4.85E-02 |
|  | Fc gamma R-mediated phagocytosis | KEGG:04666 | 9 | 2.3 | 4.85E-02 |

**Supplementary Table 8.** Genes involved in the mTOR pathway from the PFC_M23 module

| Gene Symbol | Gene Name |
| --- | --- |
| BRAF | B-Raf proto-oncogene, serine/threonine kinase(BRAF) |
| MAP2K1 | mitogen-activated protein kinase kinase 1(MAP2K1) |
| WNT5B | Wnt family member 5B(WNT5B) |
| KRAS | KRAS proto-oncogene, GTPase(KRAS) |
| LAMTOR3 | late endosomal/lysosomal adaptor, MAPK and MTOR activator 3(LAMTOR3) |
| PRKCB | protein kinase C beta(PRKCB) |
| ATP6V1A | ATPase H+ transporting V1 subunit A(ATP6V1A) |
| ATP6V1B2 | ATPase H+ transporting V1 subunit B2(ATP6V1B2) |
| RRAGA | Ras related GTP binding A(RRAGA) |
| PIK3R1 | phosphoinositide-3-kinase regulatory subunit 1(PIK3R1) |
| AKT3 | AKT serine/threonine kinase 3(AKT3) |
| ATP6V1C1 | ATPase H+ transporting V1 subunit C1(ATP6V1C1) |
| SESN2 | sestrin 2(SESN2) |
| ATP6V1G2 | ATPase H+ transporting V1 subunit G2(ATP6V1G2) |
| ATP6V1D | ATPase H+ transporting V1 subunit D(ATP6V1D) |
| EIF4B | eukaryotic translation initiation factor 4B(EIF4B) |
| WNT4 | Wnt family member 4(WNT4) |
| RRAGC | Ras related GTP binding C(RRAGC) |
| EIF4E | eukaryotic translation initiation factor 4E(EIF4E) |

**Supplementary Table 9.**  Consensus genes between mTOR pathway-related modules and cell type specific markers

| Module | Ast (n=244) | Endo (n=175) | Mic (n=284) | Neu (n=185) | Oli (n=258) |
| --- | --- | --- | --- | --- | --- |
| PFC_M4 | 1 (P=1) | 17 (P=1.2e-14) | 7 (P=0.02) | 0 (P=1) | 1 (P=0.92) |
| PFC_M5 | 27 (P=0.05) | 12 (P=0.7) | 12 (P=0.99) | 0 (P=1) | 4 (P=1) |
| PFC_M23 | 4 (P=0.99) | 2 (P=0.96) | 10 (P=0.86) | 100 (P<2.2e-16) | 10 (P=0.78) |

Ast, astrocyte; Endo, endothelial cells; Mic, microglia; Neu, neuron; Oli, oligodendrocytes

**Supplementary Table 10.**  Biological processes enriched in the STAT3-target genes from PFC_M4

|  | **term_name** | **term_id** | **count** | **Fold Enrichment** | **Adjusted p_value** |
| --- | --- | --- | --- | --- | --- |
| STAT3 | response to cytokine | GO:0034097 | 32 | 4.8 | 5.29E-11 |
|  | cellular response to cytokine stimulus | GO:0071345 | 31 | 5.0 | 5.29E-11 |
|  | immune system process | GO:0002376 | 49 | 2.8 | 6.75E-10 |
|  | cytokine-mediated signaling pathway | GO:0019221 | 25 | 5.7 | 6.75E-10 |
|  | cell death | GO:0008219 | 40 | 3.2 | 2.35E-09 |
|  | defense response | GO:0006952 | 35 | 3.6 | 3.33E-09 |
|  | response to stress | GO:0006950 | 54 | 2.4 | 4.16E-09 |
|  | inflammatory response | GO:0006954 | 23 | 5.4 | 1.11E-08 |
|  | regulation of cell death | GO:0010941 | 33 | 3.4 | 3.70E-08 |
|  | regulation of apoptotic process | GO:0042981 | 31 | 3.5 | 7.63E-08 |

**Supplementary Table 11.**  Biological processes enriched in the p53-target genes from PFC_M5

|  | **term_name** | **term_id** | **count** | **Fold Enrichment** | **Adjusted p_value** |
| --- | --- | --- | --- | --- | --- |
| p53 | SRP-dependent cotranslational protein targeting to membrane | GO:0006614 | 53 | 85.2 | 5.76E-92 |
|  | cotranslational protein targeting to membrane | GO:0006613 | 53 | 81.0 | 1.31E-90 |
|  | protein targeting to ER | GO:0045047 | 53 | 74.4 | 4.37E-88 |
|  | nuclear-transcribed mRNA catabolic process, nonsense-mediated decay | GO:0000184 | 54 | 68.3 | 2.10E-87 |
|  | establishment of protein localization to endoplasmic reticulum | GO:0072599 | 53 | 71.8 | 3.31E-87 |
|  | protein localization to endoplasmic reticulum | GO:0070972 | 53 | 58.9 | 2.00E-81 |
|  | viral transcription | GO:0019083 | 54 | 46.8 | 1.03E-76 |
|  | translational initiation | GO:0006413 | 55 | 43.3 | 3.50E-76 |
|  | viral gene expression | GO:0019080 | 54 | 42.7 | 2.49E-74 |
|  | nuclear-transcribed mRNA catabolic process | GO:0000956 | 54 | 39.5 | 2.96E-72 |

**Supplementary Table 12.** Differentially expressed genes in the PFC in BPD common to both the discovery and replication study.

**Up-regulated genes**

TXNDC11 RBM15 ARFRP1 DNPEP UBAC2 ASB6 FADS3 PRPF3 SMCHD1 GAN CLUH ALMS1 PHC3 ACVR1B RECQL5 SMC5 FLVCR1 G2E3 BRWD3 CEP57L1 RCOR1 ZNF548 MAP2K6 ZRANB3 CACFD1 IFFO2 PLA2G15 GRAMD1A DAGLB KLHL15 EML4 CRY2 PTPN13 FNDC3B ELL SIDT2 TRIM52 DNAAF5 PRKAB1 JMJD1C SELENON POU2F1 DGKD SMURF2 NIM1K ZDHHC18 ZNF460 DONSON UBOX5 MEGF11 ZNF761 PPAT MCCC2 S100PBP FBXL12 MTF2 THUMPD3-AS1 CCNF CHD2 ECE1 GNA12 PHTF2 GNAI3 CALCOCO2 CHST1 YTHDF1 ZNF516 SNX33 SELENOO SMG1 LPIN1 UBR5 ABHD14B LIMD1 KRBOX4 FRMD8 STAT3 AC097103.2 FUT1 GLIS2 TAZ TBC1D10A MLXIPL PUS10 DNMBP KIF9-AS1 C15orf39 AC064836.3 USP3 CARD19 BMP1 ASIC3 LINC00511 PPARG GTF2IRD1 GINS2 AL117335.1 SMARCD2 DRAIC CYBC1 NR6A1 TRPV1 TEF AL391244.2 ROBO3 CCDC61 CNTD1 TRAIP PSMG4 MYT1 ABCA1 PLAGL2 RABGEF1 MLXIP TTN TRIM25 TMEM39A MPP3 FGF14-AS2 AF117829.1 UPP2 ELMSAN1 AL031118.1 DGAT2 ARSD CSKMT RREB1 EZH2 AP000813.1 TUFT1 SCLY XRN1 PALM3 FAM20C AIFM2 SH3PXD2B LINC01089 AC004982.1 CAPN10-DT SLC8B1 AC005476.2 NABP1 ALDH1L2 GRAMD2A PPP1R18 MALT1 BCAT2 EVA1C LAMTOR5-AS1 AC132872.1 ASAP3 HSP90B2P EML3 ZNRF3 AC021078.1 MEX3A SCARA3 NXN SLC38A6 SNHG5 KCNQ1OT1 AC003102.1 LIMS1 SCML1 AC135506.1 LEAP2 AOC2 MOV10 ELK1 SNHG3 MOB3C HAUS8 LINC01481 LONRF3 PRSS23 CHSY1 GHR C5orf56 PRKAB2 AC025181.2 LINC-PINT LINC00174 GPR156 AC010976.1 LINC00092 TNIP2 LMBR1L NKTR STAT5A WNT4 PLIN2 GLIS3 RHBDF1 AC009407.1 AL133243.2 PVT1 EIF4EBP1 ASIC4 MDK DDIT3 ZFR2 PHLDB3 PIGA AL137025.1 AC010618.3 SH3BP2 BCL6 SNAP23 ZNF831 DHX58 TPCN2 CEP95 U73166.1 FZD4 ABCD4 FAM71F2 PLD2 TNFRSF10B JAK3 DOCK6 AC116913.1 ERBB2 CEBPB RASD1 HEXA NFIL3 HDAC7 FSTL1 PTK6 TRAF3IP2 IL15RA MAP2K3 ST20 MYC DUSP9 SHMT1 ZNF442 FCHSD1 ARRDC3 RFX4 SLC25A21-AS1 RNF43 STAC3 ANKRD18A AL359510.2 AC002553.2 PML CYTOR CFP ZNF385C LMNA FERMT3 CHST3 NFKB2 VASH2 PLOD2 AL391684.1 ACSL5 PHF21B AL117339.4 AC007614.1 RAB34 AL157700.1 SERPING1 DLGAP1-AS1 SLC7A2 TNFSF9 AC009283.1 MTHFD2 ALDH1L1 MRVI1 AC027290.2 ZNF433-AS1 LFNG MASP1 CRISPLD2 TLCD1 SMTN LCN12 AC131212.3 RGL3 MLKL RELT TES DTX3L PBX4 PLK5 N4BP2 MALAT1 LIMK2 PIM1 CAPN9 PRORSD1P DUOX1 RALGDS LTB4R CD40 SLC44A3-AS1 PRR26 MPZ SEMA4B CLIC1 EFNA1 SEMA3F TCEA3 PER1 PXN NID1 FGF2 MC1R DSC2 SBNO2 RDH10 GGT5 IRF7 RFLNB KCNE4 TIMP1 SLC4A5 SLC39A14 TNFRSF1A TCF7 PLEKHG2 PLEKHA4 TUBB6 PLK1 PDLIM4 SDS AC139100.2 IFITM3 MYBPHL FAM198A SDC4 THAP9-AS1 F3 C1R COL4A2 SERPINB1 TIPARP ADAM8 OXTR PLAUR CRISPLD1 SLC2A4 CLCF1 HIF3A AC010615.2 OSGIN1 ACKR1 TFAP2E GLI2 PXDC1 APOL6 AC008537.3 TNFRSF10D TEAD4 LOXL2 ICAM1 ITPKC ADAMTS2 ADIRF-AS1 GADD45A BAALC-AS1 RRAD ADAMTS9-AS1 SLC16A3 GNB3 HS3ST3B1 GPR4 HMOX1 IRF1 EDN1 MAFF PPP1R3B ARID5A PLSCR1 NGFR COL4A1 BAG3 IL4R HELZ2 TEAD3 NEAT1 FAM189A2 TRIP10 CDK2 HBA2 EPHA2 IL1R1 ZIC1 GABRE CHI3L1 MT1M EMP1 IFITM2 ADAMTS9 OSMR YBX3 KIAA0040 DEPP1 C11orf96 SLC11A1 MT1X CDKN1A ANGPTL4 GADD45B HAMP HILPDA SOCS

**Down-regulated genes**

MTND1P23 NEUROD6 SST LINC02192 KRT5 VGF HMGB1P6 CRHBP CRH PLCH1 LRRC53 ABCG2 ATOH7 LGI2 KCNK1 ABCC12 CCND1 LINC01202 CPED1 LINC00507 TMEFF2 EDN3 RGS4 PCSK1 CCDC184 AL391845.2 TMEM155 PTENP1 NXPH2 AC008780.2 CHCHD4 SYNDIG1 SERTAD4 HR OSBPL3 RCSD1 LINC01140 TRIB2 IGFBP6 CCKBR FBXO9 DPH3 HAPLN1 USP27X-AS1 AGMAT ARHGAP20 ELAVL2 LRRN3 CFAP57 AC017100.1 VSTM2B NDUFAF4 GPR85 GFRA2 IFT57 FSCN1 NAP1L5 SPRN RAB15 HSPA8 BEX5 DHCR24 NOV NRN1 ATP1B1 CMAS ZNF252P RIOK1 C14orf119 PFDN4 VBP1 ACAT2 SYT12 PHYHIPL ARMCX2 MKKS C1QL3 AP1S2 ELMO1 PPID C3orf14 GNG4 CPLX1 AL137003.2 OTOGL SLC8A3 COPS4 PGBD4 MAS1 RASSF3 EIF4G2 TIMM17A NUAK1 HPRT1 HOMER1 NDRG3 UBLCP1 AC120049.1 CAP2 BEND5 CHMP5 CAB39L PLS3 CACNB1 ATP5F1B HECA MEAF6 AC007405.3 MLLT11 RCAN2 NRSN1 MOAP1 PSMA4 NETO2 SERPINI1 ATL1 GABRA1 DIRAS2 DCLK1 RTN4 LSM12 STX7 ETS2 ADO LDHA SLC39A10 FAM173A COPS7A VPS35 SNAP25 SPTSSB MTX2 CEP19 MAGEH1 CHGB GOT1 C3orf80 TRIAP1 BZW1 PLPBP PFN2 ARMCX3 TMEM183A RANBP6 TMEM271 MDH1 IDH3A ELAVL4 CHAMP1 UBE2N DYNC1LI1 G3BP2 OXR1 NTN4 BASP1 TDRD7 RAB14 ATP6V1A FHL2 BTN2A1 NSF TPI1P1 SUCLA2 PSMC1 NCALD FAM216A ZNF804A CDK14 OAT BTBD10 RNF144A-AS1 LYSMD2 RHEB EIF4A2 ATP6V1D PCMT1 RPH3A OLFM3 ZNF576 PAIP2 GABRG3 MFSD6 UBL4A PLCL2 PRKACB KIFAP3 PPM1H FAM49A METAP1 GLRB OPTN EMC4 HPF1 ABHD10 PSMD2 UBE2E3 PCSK2 ATP6V1B2 MRPL3 ISCA1 MRPS22 MAP2K4 AL928654.1 PAK1 GAP43 DDX1 DLAT TIPRL ACTR6 DENR DNAJC6 MORF4L2 TMEM151B RAB2A FXR2 DNAJC25 VDAC1 HCN2 SLC25A14 SSB C1orf43 GUCY1B1 GPRASP2 RABEPK REEP1 PIGX TMEM19 UBE2V2 FASTKD5 USO1 YWHAB MAFG MRPL9 ELMOD1 FBXO45 MORF4L1 ANAPC13 MMADHC CLTC USP27X DDX50 SLC30A9 TMEM69 VWC2 GLCE PLRG1 EIF2S1 LMO4 CUL2 CEP41 VPS50 RAB11A C1GALT1C1 ZNF181 PLEKHB2 GDPD1 ITFG1 CUL3 VDAC3 SEPT7 MORF4L1P1 RAB6A DIRAS1 ADSS RPS6KA3 CFAP36 SUMO1 RBM18 R3HDM2 KIF21A BRI3BP DNAJC28 SLC38A1 SEC61A2 TVP23B FAM49B CXXC5 SRSF6 VDAC2 USP16 CTCF VPS41 PPP1R11 NKIRAS1 EI24 HEXIM1 GRSF1 PRMT2 RPRD1A MTIF2 PPP2R5E FAHD1 CLINT1 VKORC1L1 MRPS10 PIK3CB RNF19B DNAJC8 TBCC RAB1A NARS PSME3 ZNF180 PSMD14 WDR47 ATG4D DNM1L B3GALNT1 FITM2 UXS1 HNRNPAB EPB41L3 TM9SF2 PJA1 FAR2 SERINC3 CNOT7 ZFP64 MAPRE2 UBE2Q1 TERF2IP YIPF5 PINK1 LDB2 SCRN3 JKAMP DCK CAP1 EPS15 UFM1 TBK1 ATXN10 USP14 MFF PPP3CB ARCN1 MTERF3 GOLPH3 RRAGA ANAPC7 ZC3H15 PPME1 GARS MRM2 CMPK1 JCAD TUSC2 FAM122A CHSY3 STIM2 GBA SNX12 UBFD1 LCMT2 CLCN4 KCMF1 MAPK9 PITPNA WASHC2C IARS2 DIS3L PANK1 ARPC2 B4GALNT1 ING3 RSL1D1 UBA2 TAF5 FBXW7 SPRYD7 NT5C3B OSBPL1A BIRC2 DLD NOLC1 SKP1 COPA LEO1 NECAP1 FAM98B FOPNL AFTPH EPM2A HSPA9 RAB2B PPT1 PAIP1 STK24 GHITM CREBL2 AKT3 FKBP3 WSB2 KIF1BP XRCC6 STAU2 IARS TAX1BP1 NCOA4 PSMD1 APP COQ10A CD47 MED19 DUSP3 TCOF1 IPO8 NIPA2 EIF4E LONRF2 TRIM44 SH3KBP1 EIF3J ZFP91 CSDE1 ZFYVE9 RAB35 KPNA6 BCAP29 MSL3 UQCRC2 PARG SNX27 JRKL PAK1IP1 NPEPPS RAB12 KLHL12 CAPRIN1 CBX3 YME1L1 SRPRA KLHDC2 HDGFL3 ATG5 SPIN1 SUPT16H TRAPPC8 GFM1 MFN2 VPS36 SRRD HARS SIRT5 RPAP3 ARF6 UTRN ABCF2 TSPAN5 ELP1 KLHL42 NDUFAF6 HNRNPL SLTM SRPK2 EIF4G3 GPBP1L1 TM7SF3 GNB1 HEATR5B SSRP1 PPP2R5C GANAB CEBPZ WAC MBD4 WIPF2 UBE3C ESYT2 SUPT7L

**Supplementary Table 13.**  KEGG pathways enriched in the up-regulated genes common to both the discovery and replication study in BPD.

| **Term_name** | **Term_id** | **Count** | **Fold Enrichment** | **Adjusted p_value** |
| --- | --- | --- | --- | --- |
| Valine, leucine and isoleucine biosynthesis | KEGG:00290 | 2 | 22.8 | 3.15E-02 |
| One carbon pool by folate | KEGG:00670 | 4 | 9.1 | 1.45E-02 |
| Thyroid cancer | KEGG:05216 | 6 | 7.4 | 1.31E-02 |
| Circadian rhythm | KEGG:04710 | 4 | 5.9 | 3.94E-02 |
| Endometrial cancer | KEGG:05213 | 7 | 5.5 | 1.31E-02 |
| Non-small cell lung cancer | KEGG:05223 | 8 | 5.1 | 1.31E-02 |
| Basal cell carcinoma | KEGG:05217 | 7 | 5.1 | 1.38E-02 |
| Acute myeloid leukemia | KEGG:05221 | 7 | 4.8 | 1.45E-02 |
| Adipocytokine signaling pathway | KEGG:04920 | 7 | 4.6 | 1.45E-02 |
| Pancreatic cancer | KEGG:05212 | 7 | 4.2 | 1.98E-02 |
| NF-kappa B signaling pathway | KEGG:04064 | 9 | 4.0 | 1.38E-02 |
| Insulin resistance | KEGG:04931 | 9 | 3.8 | 1.45E-02 |
| p53 signaling pathway | KEGG:04115 | 6 | 3.7 | 4.17E-02 |
| TNF signaling pathway | KEGG:04668 | 9 | 3.7 | 1.45E-02 |
| AGE-RAGE signaling pathway in diabetic complications | KEGG:04933 | 8 | 3.6 | 1.98E-02 |
| FoxO signaling pathway | KEGG:04068 | 10 | 3.5 | 1.45E-02 |
| Small cell lung cancer | KEGG:05222 | 7 | 3.5 | 3.80E-02 |
| Breast cancer | KEGG:05224 | 10 | 3.1 | 1.98E-02 |
| JAK-STAT signaling pathway | KEGG:04630 | 11 | 3.1 | 1.45E-02 |
| Gastric cancer | KEGG:05226 | 10 | 3.1 | 1.98E-02 |
| Epstein-Barr virus infection | KEGG:05169 | 13 | 3.0 | 1.38E-02 |
| Signaling pathways regulating pluripotency of stem cells | KEGG:04550 | 9 | 2.9 | 3.81E-02 |
| Transcriptional misregulation in cancer | KEGG:05202 | 12 | 2.9 | 1.58E-02 |
| Hepatitis B | KEGG:05161 | 10 | 2.8 | 3.15E-02 |
| Hepatocellular carcinoma | KEGG:05225 | 10 | 2.7 | 3.59E-02 |
| Pathways in cancer | KEGG:05200 | 31 | 2.7 | 9.80E-05 |
| MAPK signaling pathway | KEGG:04010 | 17 | 2.6 | 1.31E-02 |
| Lipid and atherosclerosis | KEGG:05417 | 12 | 2.6 | 3.15E-02 |
| Human T-cell leukemia virus 1 infection | KEGG:05166 | 12 | 2.5 | 3.36E-02 |
| Human cytomegalovirus infection | KEGG:05163 | 12 | 2.5 | 3.59E-02 |
| Proteoglycans in cancer | KEGG:05205 | 11 | 2.4 | 4.21E-02 |
| Cytokine-cytokine receptor interaction | KEGG:04060 | 14 | 2.2 | 4.17E-02 |
| PI3K-Akt signaling pathway | KEGG:04151 | 16 | 2.1 | 3.97E-02 |

**Supplementary Table 14.**  The differentially expressed genes involved in the mTOR pathway common to both the discovery and replication study

| Gene symbol | Gene name | Regulation |
| --- | --- | --- |
| CAB39L | protein kinase AMP-activated catalytic subunit alpha 2 | Down |
| PIK3CB | protein kinase C beta | Down |
| RPS6KA3 | 3-phosphoinositide dependent protein kinase 1 | Down |
| RHEB | ribosomal protein S6 | Down |
| EIF4E | Ras related GTP binding A | Down |
| RRAGA | Ras homolog enriched in brain | Down |
| AKT3 | AKT serine/threonine kinase 3 | Down |
| ATP6V1D | ATPase H+ transporting V1 subunit D(ATP6V1D) | Down |
| ATP6V1A | ATPase H+ transporting V1 subunit A(ATP6V1A) | Down |
| ATP6V1B2 | ATPase H+ transporting V1 subunit B2(ATP6V1B2) | Down |
| EIF4EBP1 | eukaryotic translation initiation factor 4E | Up |

**Supplementary Table 15.**  KEGG pathways enriched in the down-regulated genes common to both the discovery and replication study in BPD.

| **Term_name** | **Term_id** | **Count** | **Fold Enrichment** | **Adjusted p_value** |
| --- | --- | --- | --- | --- |
| Citrate cycle (TCA cycle) | KEGG:00020 | 5 | 7.4 | 1.64E-02 |
| Proteasome | KEGG:03050 | 6 | 5.8 | 1.64E-02 |
| Mitophagy - animal | KEGG:04137 | 7 | 4.3 | 2.60E-02 |
| Synaptic vesicle cycle | KEGG:04721 | 7 | 4.0 | 3.34E-02 |
| Ubiquitin mediated proteolysis | KEGG:04120 | 12 | 3.8 | 1.13E-02 |
| Spinocerebellar ataxia | KEGG:05017 | 12 | 3.7 | 1.13E-02 |
| Autophagy - animal | KEGG:04140 | 11 | 3.5 | 1.51E-02 |
| AMPK signaling pathway | KEGG:04152 | 9 | 3.3 | 2.98E-02 |
| Yersinia infection | KEGG:05135 | 10 | 3.3 | 2.50E-02 |
| Oocyte meiosis | KEGG:04114 | 9 | 3.0 | 4.24E-02 |
| mTOR signaling pathway | KEGG:04150 | 10 | 2.9 | 4.02E-02 |
| Shigellosis | KEGG:05131 | 16 | 2.9 | 1.15E-02 |
| Endocytosis | KEGG:04144 | 16 | 2.8 | 1.15E-02 |
| Parkinson disease | KEGG:05012 | 16 | 2.7 | 1.51E-02 |
| Human T-cell leukemia virus 1 infection | KEGG:05166 | 13 | 2.6 | 2.82E-02 |
| Prion disease | KEGG:05020 | 16 | 2.6 | 1.63E-02 |
| Alzheimer disease | KEGG:05010 | 19 | 2.2 | 2.50E-02 |
| Pathways of neurodegeneration - multiple diseases | KEGG:05022 | 21 | 2.0 | 3.97E-02 |

**Supplementary Table 16.** Correlation coefficients between co-expression modules from the replication data set and BPD or descriptive variables

| **Module** | **BP** | **Age** | **Sex** | **Brain pH** | **PMI** |
| --- | --- | --- | --- | --- | --- |
| R_PFC_M1 | N.S | N.S | N.S | N.S | N.S |
| R_PFC_M2 | N.S | N.S | N.S | N.S | N.S |
| R_PFC_M3 | 0.25 | N.S | N.S | -0.31 | N.S |
| R_PFC_M4 | 0.26 | N.S | N.S | N.S | N.S |
| R_PFC_M5 | N.S | -0.49 | N.S | 0.25 | N.S |
| R_PFC_M6 | N.S | N.S | N.S | 0.33 | N.S |
| R_PFC_M7 | -0.25 | N.S | N.S | 0.27 | N.S |
| R_PFC_M8 | N.S | N.S | N.S | 0.24 | N.S |
| R_PFC_M9 | -0.23 | -0.31 | N.S | N.S | N.S |
| R_PFC_M10 | N.S | N.S | N.S | 0.29 | N.S |
| R_PFC_M11 | N.S | -0.27 | N.S | 0.32 | N.S |
| R_PFC_M12 | N.S | N.S | -0.90 | N.S | N.S |
| R_PFC_M13 | N.S | N.S | N.S | N.S | N.S |
| R_PFC_M14 | N.S | N.S | N.S | -0.31 | N.S |
| R_PFC_M15 | 0.28 | N.S | N.S | -0.25 | N.S |
| R_PFC_M16 | N.S | N.S | N.S | 0.26 | N.S |
| R_PFC_M17 | N.S | N.S | N.S | N.S | N.S |
| R_PFC_M18 | N.S | N.S | N.S | N.S | N.S |
| R_PFC_M19 | N.S | N.S | N.S | N.S | N.S |
| R_PFC_M20 | N.S | N.S | N.S | N.S | N.S |

**Supplementary Table 17.**  Number of consensus genes between the BPD-associated modules and the differentially expressed genes from the replication dataset

| Module | Down regulated genes (n=2139) | Up regulated genes (n=3399) |
| --- | --- | --- |
| R_PFC_M3 | 24 (P=1) | 1039 (P<2.2e-16) |
| R_PFC_M4 | 1 (P=1) | 354 (P<2.2e-16) |
| R_PFC_M7 | 1262 (P<2.2e-16) | 364 (P=1) |
| R_PFC_M9 | 11 (P=4.0e-4) | 2 (P=0.98) |
| R_PFC_M15 | 1 (P=1) | 175 (P<2.2e-16) |

**Supplementary Table 18.**  KEGG pathways enriched in the genes common to both PFC_M4 and the R_PFC_M15 module.

| **Term_name** | **Term_id** | **Count** | **Fold Enrichment** | **Adjusted p_value** |
| --- | --- | --- | --- | --- |
| TNF signaling pathway | KEGG:04668 | 10 | 15.9 | 6.57E-08 |
| Epstein-Barr virus infection | KEGG:05169 | 9 | 8.1 | 9.59E-05 |
| JAK-STAT signaling pathway | KEGG:04630 | 8 | 8.8 | 1.46E-04 |
| Small cell lung cancer | KEGG:05222 | 6 | 11.6 | 3.59E-04 |
| Kaposi sarcoma-associated herpesvirus infection | KEGG:05167 | 8 | 7.4 | 3.59E-04 |
| Hepatitis C | KEGG:05160 | 7 | 7.9 | 4.26E-04 |
| Pathways in cancer | KEGG:05200 | 12 | 4.0 | 4.26E-04 |
| Human T-cell leukemia virus 1 infection | KEGG:05166 | 8 | 6.6 | 4.26E-04 |
| NF-kappa B signaling pathway | KEGG:04064 | 6 | 10.5 | 4.26E-04 |
| Thyroid cancer | KEGG:05216 | 4 | 19.2 | 8.11E-04 |
| Chronic myeloid leukemia | KEGG:05220 | 5 | 11.7 | 9.01E-04 |
| Transcriptional misregulation in cancer | KEGG:05202 | 7 | 6.5 | 1.12E-03 |
| Cellular senescence | KEGG:04218 | 6 | 6.8 | 2.61E-03 |
| AGE-RAGE signaling pathway in diabetic complications | KEGG:04933 | 5 | 8.9 | 2.61E-03 |
| Hepatitis B | KEGG:05161 | 6 | 6.6 | 2.74E-03 |
| Th17 cell differentiation | KEGG:04659 | 5 | 8.5 | 2.74E-03 |
| Endometrial cancer | KEGG:05213 | 4 | 12.3 | 2.81E-03 |
| Salmonella infection | KEGG:05132 | 7 | 5.0 | 3.84E-03 |
| Acute myeloid leukemia | KEGG:05221 | 4 | 10.6 | 4.38E-03 |
| Adipocytokine signaling pathway | KEGG:04920 | 4 | 10.3 | 4.66E-03 |
| Cell cycle | KEGG:04110 | 5 | 7.2 | 4.71E-03 |
| Non-small cell lung cancer | KEGG:05223 | 4 | 9.9 | 4.98E-03 |
| p53 signaling pathway | KEGG:04115 | 4 | 9.7 | 5.02E-03 |
| FoxO signaling pathway | KEGG:04068 | 5 | 6.8 | 5.29E-03 |
| Pancreatic cancer | KEGG:05212 | 4 | 9.4 | 5.37E-03 |
| Apoptosis | KEGG:04210 | 5 | 6.5 | 5.78E-03 |
| Fluid shear stress and atherosclerosis | KEGG:05418 | 5 | 6.4 | 5.94E-03 |
| MAPK signaling pathway | KEGG:04010 | 7 | 4.2 | 6.62E-03 |
| Colorectal cancer | KEGG:05210 | 4 | 8.3 | 7.32E-03 |
| Gastric cancer | KEGG:05226 | 5 | 6.0 | 7.32E-03 |
| Human cytomegalovirus infection | KEGG:05163 | 6 | 4.8 | 7.47E-03 |
| PD-L1 expression and PD-1 checkpoint pathway in cancer | KEGG:05235 | 4 | 8.0 | 7.58E-03 |
| Influenza A | KEGG:05164 | 5 | 5.3 | 1.19E-02 |
| Toxoplasmosis | KEGG:05145 | 4 | 6.5 | 1.41E-02 |
| HIF-1 signaling pathway | KEGG:04066 | 4 | 6.5 | 1.41E-02 |
| Insulin resistance | KEGG:04931 | 4 | 6.6 | 1.41E-02 |
| PI3K-Akt signaling pathway | KEGG:04151 | 7 | 3.5 | 1.42E-02 |
| Pathogenic Escherichia coli infection | KEGG:05130 | 5 | 4.5 | 1.96E-02 |
| Osteoclast differentiation | KEGG:04380 | 4 | 5.7 | 2.10E-02 |
| Basal cell carcinoma | KEGG:05217 | 3 | 8.5 | 2.10E-02 |
| Proteoglycans in cancer | KEGG:05205 | 5 | 4.3 | 2.20E-02 |
| Central carbon metabolism in cancer | KEGG:05230 | 3 | 7.6 | 2.56E-02 |
| Prolactin signaling pathway | KEGG:04917 | 3 | 7.6 | 2.56E-02 |
| Lipid and atherosclerosis | KEGG:05417 | 5 | 4.2 | 2.56E-02 |
| Melanoma | KEGG:05218 | 3 | 7.4 | 2.71E-02 |
| Glioma | KEGG:05214 | 3 | 7.1 | 2.96E-02 |
| Breast cancer | KEGG:05224 | 4 | 4.8 | 3.11E-02 |
| Hepatocellular carcinoma | KEGG:05225 | 4 | 4.3 | 4.61E-02 |

**Supplementary Table 19.**  KEGG pathways enriched in the genes common to both PFC_M23 and the R_PFC_M7 module.

| **Term_name** | **Term_id** | **Count** | **Fold Enrichment** | **Adjusted p_value** |
| --- | --- | --- | --- | --- |
| cGMP-PKG signaling pathway | KEGG:04022 | 17 | 3.86 | 2.35E-04 |
| Dopaminergic synapse | KEGG:04728 | 15 | 4.32 | 2.35E-04 |
| Synaptic vesicle cycle | KEGG:04721 | 11 | 5.32 | 3.12E-04 |
| Spinocerebellar ataxia | KEGG:05017 | 15 | 3.99 | 3.12E-04 |
| GnRH signaling pathway | KEGG:04912 | 12 | 4.87 | 3.12E-04 |
| Endocrine and other factor-regulated calcium reabsorption | KEGG:04961 | 9 | 6.41 | 3.98E-04 |
| Alzheimer disease | KEGG:05010 | 25 | 2.56 | 4.66E-04 |
| Epithelial cell signaling in Helicobacter pylori infection | KEGG:05120 | 10 | 5.39 | 4.66E-04 |
| Oocyte meiosis | KEGG:04114 | 13 | 3.83 | 9.63E-04 |
| Phosphatidylinositol signaling system | KEGG:04070 | 11 | 4.28 | 1.07E-03 |
| mTOR signaling pathway | KEGG:04150 | 14 | 3.43 | 1.07E-03 |
| Adrenergic signaling in cardiomyocytes | KEGG:04261 | 14 | 3.52 | 1.07E-03 |
| Inflammatory mediator regulation of TRP channels | KEGG:04750 | 11 | 4.24 | 1.07E-03 |
| Pathways of neurodegeneration - multiple diseases | KEGG:05022 | 28 | 2.22 | 1.07E-03 |
| ErbB signaling pathway | KEGG:04012 | 10 | 4.49 | 1.11E-03 |
| Long-term potentiation | KEGG:04720 | 9 | 5.07 | 1.11E-03 |
| Insulin signaling pathway | KEGG:04910 | 13 | 3.58 | 1.11E-03 |
| T cell receptor signaling pathway | KEGG:04660 | 11 | 4.03 | 1.25E-03 |
| Parkinson disease | KEGG:05012 | 18 | 2.73 | 1.47E-03 |
| Long-term depression | KEGG:04730 | 8 | 5.12 | 1.83E-03 |
| Salivary secretion | KEGG:04970 | 10 | 4.15 | 1.83E-03 |
| VEGF signaling pathway | KEGG:04370 | 8 | 5.12 | 1.83E-03 |
| Human T-cell leukemia virus 1 infection | KEGG:05166 | 16 | 2.80 | 2.24E-03 |
| Oxytocin signaling pathway | KEGG:04921 | 13 | 3.19 | 2.43E-03 |
| Cellular senescence | KEGG:04218 | 13 | 3.14 | 2.60E-03 |
| Apelin signaling pathway | KEGG:04371 | 12 | 3.33 | 2.60E-03 |
| Sphingolipid signaling pathway | KEGG:04071 | 11 | 3.49 | 2.73E-03 |
| Progesterone-mediated oocyte maturation | KEGG:04914 | 10 | 3.81 | 2.73E-03 |
| Growth hormone synthesis, secretion and action | KEGG:04935 | 11 | 3.49 | 2.73E-03 |
| AGE-RAGE signaling pathway in diabetic complications | KEGG:04933 | 10 | 3.77 | 2.73E-03 |
| Vibrio cholerae infection | KEGG:05110 | 7 | 5.28 | 2.76E-03 |
| Chagas disease | KEGG:05142 | 10 | 3.74 | 2.76E-03 |
| C-type lectin receptor signaling pathway | KEGG:04625 | 10 | 3.63 | 3.40E-03 |
| Amphetamine addiction | KEGG:05031 | 8 | 4.38 | 3.46E-03 |
| Renin secretion | KEGG:04924 | 8 | 4.38 | 3.46E-03 |
| Gap junction | KEGG:04540 | 9 | 3.86 | 3.84E-03 |
| GABAergic synapse | KEGG:04727 | 9 | 3.82 | 4.06E-03 |
| Calcium signaling pathway | KEGG:04020 | 16 | 2.53 | 4.20E-03 |
| Relaxin signaling pathway | KEGG:04926 | 11 | 3.22 | 4.20E-03 |
| Collecting duct acid secretion | KEGG:04966 | 5 | 6.99 | 4.27E-03 |
| Serotonergic synapse | KEGG:04726 | 10 | 3.37 | 4.93E-03 |
| Glutamatergic synapse | KEGG:04724 | 10 | 3.31 | 5.30E-03 |
| Pancreatic cancer | KEGG:05212 | 8 | 3.97 | 5.30E-03 |
| Gastric acid secretion | KEGG:04971 | 8 | 3.97 | 5.30E-03 |
| Salmonella infection | KEGG:05132 | 16 | 2.42 | 5.56E-03 |
| Autophagy - animal | KEGG:04140 | 11 | 3.03 | 5.90E-03 |
| Aldosterone synthesis and secretion | KEGG:04925 | 9 | 3.47 | 6.45E-03 |
| Neurotrophin signaling pathway | KEGG:04722 | 10 | 3.17 | 6.75E-03 |
| Thyroid hormone signaling pathway | KEGG:04919 | 10 | 3.12 | 7.36E-03 |
| Human immunodeficiency virus 1 infection | KEGG:05170 | 14 | 2.52 | 7.36E-03 |
| Carbohydrate digestion and absorption | KEGG:04973 | 6 | 4.82 | 7.49E-03 |
| GnRH secretion | KEGG:04929 | 7 | 4.13 | 7.52E-03 |
| Pancreatic secretion | KEGG:04972 | 9 | 3.33 | 7.60E-03 |
| cAMP signaling pathway | KEGG:04024 | 14 | 2.45 | 8.87E-03 |
| Synthesis and degradation of ketone bodies | KEGG:00072 | 3 | 11.32 | 9.03E-03 |
| Fc epsilon RI signaling pathway | KEGG:04664 | 7 | 3.94 | 9.03E-03 |
| Insulin secretion | KEGG:04911 | 8 | 3.51 | 9.03E-03 |
| Colorectal cancer | KEGG:05210 | 8 | 3.51 | 9.03E-03 |
| Renal cell carcinoma | KEGG:05211 | 7 | 3.88 | 9.40E-03 |
| Cardiac muscle contraction | KEGG:04260 | 8 | 3.47 | 9.40E-03 |
| Shigellosis | KEGG:05131 | 15 | 2.30 | 9.91E-03 |
| Cholinergic synapse | KEGG:04725 | 9 | 3.01 | 1.32E-02 |
| Glioma | KEGG:05214 | 7 | 3.52 | 1.53E-02 |
| Endocrine resistance | KEGG:01522 | 8 | 3.18 | 1.53E-02 |
| Fc gamma R-mediated phagocytosis | KEGG:04666 | 8 | 3.14 | 1.61E-02 |
| Circadian entrainment | KEGG:04713 | 8 | 3.11 | 1.69E-02 |
| Choline metabolism in cancer | KEGG:05231 | 8 | 3.08 | 1.77E-02 |
| Lipid and atherosclerosis | KEGG:05417 | 13 | 2.29 | 1.78E-02 |
| B cell receptor signaling pathway | KEGG:04662 | 7 | 3.34 | 1.90E-02 |
| MAPK signaling pathway | KEGG:04010 | 16 | 2.05 | 1.92E-02 |
| Melanogenesis | KEGG:04916 | 8 | 2.99 | 1.98E-02 |
| Kaposi sarcoma-associated herpesvirus infection | KEGG:05167 | 12 | 2.35 | 1.98E-02 |
| Vasopressin-regulated water reabsorption | KEGG:04962 | 5 | 4.29 | 2.17E-02 |
| Prion disease | KEGG:05020 | 15 | 2.07 | 2.17E-02 |
| Endocytosis | KEGG:04144 | 14 | 2.10 | 2.54E-02 |
| Type II diabetes mellitus | KEGG:04930 | 5 | 4.10 | 2.54E-02 |
| Glucagon signaling pathway | KEGG:04922 | 8 | 2.82 | 2.61E-02 |
| Citrate cycle (TCA cycle) | KEGG:00020 | 4 | 5.03 | 2.64E-02 |
| Diabetic cardiomyopathy | KEGG:05415 | 12 | 2.23 | 2.64E-02 |
| Ras signaling pathway | KEGG:04014 | 13 | 2.12 | 2.84E-02 |
| Vascular smooth muscle contraction | KEGG:04270 | 9 | 2.55 | 2.89E-02 |
| Oxidative phosphorylation | KEGG:00190 | 9 | 2.55 | 2.89E-02 |
| Cysteine and methionine metabolism | KEGG:00270 | 5 | 3.85 | 3.03E-02 |
| Central carbon metabolism in cancer | KEGG:05230 | 6 | 3.23 | 3.31E-02 |
| Prolactin signaling pathway | KEGG:04917 | 6 | 3.23 | 3.31E-02 |
| Estrogen signaling pathway | KEGG:04915 | 9 | 2.48 | 3.31E-02 |
| Fluid shear stress and atherosclerosis | KEGG:05418 | 9 | 2.46 | 3.43E-02 |
| Ubiquitin mediated proteolysis | KEGG:04120 | 9 | 2.44 | 3.54E-02 |
| Carbon metabolism | KEGG:01200 | 8 | 2.60 | 3.60E-02 |
| Metabolic pathways | KEGG:01100 | 53 | 1.34 | 3.66E-02 |
| Inositol phosphate metabolism | KEGG:00562 | 6 | 3.10 | 3.77E-02 |
| Human cytomegalovirus infection | KEGG:05163 | 12 | 2.03 | 4.55E-02 |
| Alanine, aspartate and glutamate metabolism | KEGG:00250 | 4 | 4.08 | 4.59E-02 |
| Aldosterone-regulated sodium reabsorption | KEGG:04960 | 4 | 4.08 | 4.59E-02 |
| Thyroid cancer | KEGG:05216 | 4 | 4.08 | 4.59E-02 |
| Retrograde endocannabinoid signaling | KEGG:04723 | 9 | 2.29 | 4.74E-02 |
| Cell cycle | KEGG:04110 | 8 | 2.43 | 4.79E-02 |

**Supplementary Table 20.** Correlation coefficients between co-expression modules in the primary neurons and Torin1 treatment or RIN

| **Module** | **Torin1 treatment** | **RIN** |
| --- | --- | --- |
| Torin1_M1 | N.S | N.S |
| Torin1_M2 | N.S | N.S |
| Torin1_M3 | N.S | N.S |
| Torin1_M4 | N.S | N.S |
| Torin1_M5 | -0.70 | N.S |
| Torin1_M6 | -0.99 | N.S |
| Torin1_M7 | -0.70 | N.S |
| Torin1_M8 | N.S | N.S |
| Torin1_M9 | -0.71 | N.S |
| Torin1_M10 | N.S | N.S |
| Torin1_M11 | N.S | N.S |
| Torin1_M12 | N.S | N.S |
| Torin1_M13 | 0.67 | N.S |
| Torin1_M14 | 0.64 | N.S |
| Torin1_M15 | 0.96 | N.S |
| Torin1_M16 | N.S | N.S |
| Torin1_M17 | N.S | N.S |
| Torin1_M18 | N.S | N.S |
| Torin1_M19 | N.S | N.S |
| Torin1_M20 | N.S | N.S |
| Torin1_M21 | 0.63 | N.S |
| Torin1_M22 | 0.59 | N.S |
| Torin1_M23 | N.S | N.S |

N.S, not significant; RIN, RNA integrity number

**Supplementary Table 21.**  Consensus genes between the modules from Torin1-treated neurons and the differentially expressed genes

| Module | Down regulated genes (n=3956) | Up regulated genes (n=5406) |
| --- | --- | --- |
| Torin1_M5 | 244 (P=0.57) | 250 (P=1) |
| Torin1_M6 | 2317 (P<2.2e-16) | 1760 (P=0.59) |
| Torin1_M7 | 8 (P=0.56) | 18 (P=0.03) |
| Torin1_M9 | 47 (P=0.27) | 35 (P=1) |
| Torin1_M13 | 9 (P=0.96) | 21 (P=0.31) |
| Torin1_M14 | 108 (P=1) | 341 (P=1) |
| Torin1_M15 | 728 (P=1) | 1283 (P<2.2e-16) |
| Torin1_M21 | 121 (P=1) | 290 (P=0.97) |
| Torin1_M22 | 21 (P=1) | 330(P=1) |

**Supplementary Table 22.**  KEGG pathways enriched in the genes in the Torin1_M6 and Torin1_M15 modules

| **Module** | **term name** | **term id** | **count** | **Fold Enrichment** | **Adjusted p value** |
| --- | --- | --- | --- | --- | --- |
| Torin1_M6 | Cell cycle | KEGG:04110 | 71 | 2.4 | 1.58E-13 |
|  | Proteasome | KEGG:03050 | 33 | 3 | 3.20E-09 |
|  | Spliceosome | KEGG:03040 | 64 | 2.1 | 1.34E-08 |
|  | DNA replication | KEGG:03030 | 25 | 3 | 2.63E-07 |
|  | Carbon metabolism | KEGG:01200 | 55 | 1.9 | 3.84E-06 |
|  | Metabolic pathways | KEGG:01100 | 430 | 1.2 | 6.95E-06 |
|  | Alzheimer disease | KEGG:05010 | 72 | 1.7 | 7.46E-06 |
|  | RNA transport | KEGG:03013 | 67 | 1.7 | 1.36E-05 |
|  | Hepatitis B | KEGG:05161 | 64 | 1.8 | 1.78E-05 |
|  | Oocyte meiosis | KEGG:04114 | 51 | 1.8 | 3.83E-05 |
|  | Fanconi anemia pathway | KEGG:03460 | 27 | 2.4 | 3.83E-05 |
|  | Epstein-Barr virus infection | KEGG:05169 | 81 | 1.6 | 4.78E-05 |
|  | Autophagy - animal | KEGG:04140 | 57 | 1.8 | 4.78E-05 |
|  | Non-alcoholic fatty liver disease (NAFLD) | KEGG:04932 | 62 | 1.7 | 4.80E-05 |
|  | Oxidative phosphorylation | KEGG:00190 | 56 | 1.7 | 7.48E-05 |
|  | Parkinson disease | KEGG:05012 | 59 | 1.7 | 7.62E-05 |
|  | Thermogenesis | KEGG:04714 | 85 | 1.6 | 7.62E-05 |
|  | Hepatitis C | KEGG:05160 | 59 | 1.6 | 3.90E-04 |
|  | Herpes simplex virus 1 infection | KEGG:05168 | 107 | 1.4 | 3.90E-04 |
|  | Huntington disease | KEGG:05016 | 71 | 1.6 | 3.90E-04 |
|  | AGE-RAGE signaling pathway in diabetic complications | KEGG:04933 | 43 | 1.8 | 4.50E-04 |
|  | Progesterone-mediated oocyte maturation | KEGG:04914 | 39 | 1.8 | 4.70E-04 |
|  | Nucleotide excision repair | KEGG:03420 | 23 | 2.2 | 5.60E-04 |
|  | Amino sugar and nucleotide sugar metabolism | KEGG:00520 | 24 | 2.1 | 6.00E-04 |
|  | Chronic myeloid leukemia | KEGG:05220 | 34 | 1.9 | 8.30E-04 |
|  | Lysosome | KEGG:04142 | 48 | 1.7 | 8.40E-04 |
|  | FoxO signaling pathway | KEGG:04068 | 49 | 1.6 | 9.70E-04 |
|  | Cellular senescence | KEGG:04218 | 62 | 1.5 | 2.07E-03 |
|  | Protein processing in endoplasmic reticulum | KEGG:04141 | 59 | 1.5 | 2.49E-03 |
|  | mTOR signaling pathway | KEGG:04150 | 56 | 1.5 | 3.01E-03 |
|  | Steroid biosynthesis | KEGG:00100 | 12 | 2.7 | 3.05E-03 |
|  | Apoptosis | KEGG:04210 | 48 | 1.6 | 4.11E-03 |
|  | Citrate cycle (TCA cycle) | KEGG:00020 | 16 | 2.2 | 4.24E-03 |
|  | Aminoacyl-tRNA biosynthesis | KEGG:00970 | 21 | 2 | 4.24E-03 |
|  | Human papillomavirus infection | KEGG:05165 | 106 | 1.3 | 4.69E-03 |
|  | Pancreatic cancer | KEGG:05212 | 31 | 1.7 | 4.96E-03 |
|  | Homologous recombination | KEGG:03440 | 19 | 2 | 5.21E-03 |
|  | Ubiquitin mediated proteolysis | KEGG:04120 | 50 | 1.5 | 5.43E-03 |
|  | Spinocerebellar ataxia | KEGG:05017 | 37 | 1.6 | 5.44E-03 |
|  | Neurotrophin signaling pathway | KEGG:04722 | 44 | 1.6 | 5.44E-03 |
|  | Mismatch repair | KEGG:03430 | 13 | 2.4 | 5.88E-03 |
|  | One carbon pool by folate | KEGG:00670 | 11 | 2.6 | 5.99E-03 |
|  | Insulin signaling pathway | KEGG:04910 | 47 | 1.5 | 5.99E-03 |
|  | Insulin resistance | KEGG:04931 | 40 | 1.6 | 6.40E-03 |
|  | Small cell lung cancer | KEGG:05222 | 35 | 1.6 | 6.90E-03 |
|  | Longevity regulating pathway | KEGG:04211 | 34 | 1.6 | 7.17E-03 |
|  | Salmonella infection | KEGG:05132 | 70 | 1.4 | 7.64E-03 |
|  | Biosynthesis of amino acids | KEGG:01230 | 31 | 1.6 | 1.04E-02 |
|  | Glyoxylate and dicarboxylate metabolism | KEGG:00630 | 15 | 2.1 | 1.06E-02 |
|  | Colorectal cancer | KEGG:05210 | 32 | 1.6 | 1.49E-02 |
|  | Mitophagy - animal | KEGG:04137 | 25 | 1.7 | 1.72E-02 |
|  | AMPK signaling pathway | KEGG:04152 | 43 | 1.5 | 1.72E-02 |
|  | Glucagon signaling pathway | KEGG:04922 | 34 | 1.6 | 1.88E-02 |
|  | Base excision repair | KEGG:03410 | 16 | 1.9 | 2.14E-02 |
|  | Apoptosis - multiple species | KEGG:04215 | 15 | 2 | 2.14E-02 |
|  | Pyrimidine metabolism | KEGG:00240 | 23 | 1.7 | 2.28E-02 |
|  | Adipocytokine signaling pathway | KEGG:04920 | 27 | 1.6 | 2.28E-02 |
|  | MAPK signaling pathway | KEGG:04010 | 90 | 1.3 | 2.40E-02 |
|  | Circadian rhythm | KEGG:04710 | 14 | 2 | 2.77E-02 |
|  | Propanoate metabolism | KEGG:00640 | 15 | 1.9 | 2.77E-02 |
|  | Prostate cancer | KEGG:05215 | 34 | 1.5 | 3.41E-02 |
|  | Amyotrophic lateral sclerosis (ALS) | KEGG:05014 | 23 | 1.6 | 3.41E-02 |
|  | Human T-cell leukemia virus 1 infection | KEGG:05166 | 72 | 1.3 | 3.41E-02 |
|  | Valine, leucine and isoleucine degradation | KEGG:00280 | 21 | 1.7 | 3.75E-02 |
|  | RIG-I-like receptor signaling pathway | KEGG:04622 | 24 | 1.6 | 3.86E-02 |
|  | Pathways in cancer | KEGG:05200 | 146 | 1.2 | 4.75E-02 |
|  | HIF-1 signaling pathway | KEGG:04066 | 37 | 1.4 | 4.96E-02 |
| Torin1_M15 | Non-small cell lung cancer | KEGG:05223 | 20 | 2.6 | 3.47E-03 |
|  | Choline metabolism in cancer | KEGG:05231 | 26 | 2.3 | 3.47E-03 |
|  | ErbB signaling pathway | KEGG:04012 | 24 | 2.4 | 3.47E-03 |
|  | Autophagy - animal | KEGG:04140 | 31 | 1.9 | 1.37E-02 |
|  | Renal cell carcinoma | KEGG:05211 | 19 | 2.4 | 1.37E-02 |
|  | Thyroid hormone signaling pathway | KEGG:04919 | 27 | 2 | 1.51E-02 |
|  | Mitophagy - animal | KEGG:04137 | 17 | 2.3 | 1.88E-02 |
|  | Ras signaling pathway | KEGG:04014 | 44 | 1.6 | 1.88E-02 |
|  | Growth hormone synthesis, secretion and action | KEGG:04935 | 26 | 1.9 | 1.88E-02 |
|  | Glioma | KEGG:05214 | 19 | 2.2 | 1.88E-02 |
|  | GnRH signaling pathway | KEGG:04912 | 21 | 2 | 3.39E-02 |
|  | Legionellosis | KEGG:05134 | 15 | 2.3 | 3.39E-02 |
|  | EGFR tyrosine kinase inhibitor resistance | KEGG:01521 | 19 | 2 | 3.79E-02 |
|  | Endocrine resistance | KEGG:01522 | 21 | 1.9 | 3.79E-02 |
|  | Wnt signaling pathway | KEGG:04310 | 31 | 1.7 | 3.79E-02 |
|  | Long-term potentiation | KEGG:04720 | 16 | 2.2 | 3.79E-02 |
|  | Glycerophospholipid metabolism | KEGG:00564 | 22 | 1.9 | 3.79E-02 |
|  | Pancreatic cancer | KEGG:05212 | 18 | 2 | 3.79E-02 |
|  | Human papillomavirus infection | KEGG:05165 | 57 | 1.4 | 3.79E-02 |
|  | Metabolic pathways | KEGG:01100 | 206 | 1.2 | 4.17E-02 |
|  | Chronic myeloid leukemia | KEGG:05220 | 18 | 2 | 4.50E-02 |
|  | mTOR signaling pathway | KEGG:04150 | 30 | 1.7 | 4.94E-02 |

**Supplementary Table 23.**  KEGG pathways enriched in the genes common to both the PFC_M5 and Torin1_M6 modules.

| **Term_name** | **Term_id** | **Count** | **Fold Enrichment** | **Adjusted_p_value** |
| --- | --- | --- | --- | --- |
| Huntington disease | KEGG:05016 | 37 | 5.067613 | 2.08E-14 |
| Parkinson disease | KEGG:05012 | 32 | 5.386092 | 2.79E-13 |
| Prion disease | KEGG:05020 | 33 | 5.066108 | 3.02E-13 |
| Oxidative phosphorylation | KEGG:00190 | 24 | 7.562802 | 3.02E-13 |
| Thermogenesis | KEGG:04714 | 30 | 5.442925 | 6.87E-13 |
| Amyotrophic lateral sclerosis | KEGG:05014 | 37 | 4.271872 | 9.12E-13 |
| Alzheimer disease | KEGG:05010 | 33 | 3.758281 | 7.54E-10 |
| Proteasome | KEGG:03050 | 13 | 11.84428 | 7.54E-10 |
| Ribosome | KEGG:03010 | 18 | 4.93065 | 4.77E-07 |
| Non-alcoholic fatty liver disease | KEGG:04932 | 17 | 4.74986 | 1.72E-06 |
| Spliceosome | KEGG:03040 | 17 | 4.74986 | 1.72E-06 |
| Metabolic pathways | KEGG:01100 | 57 | 1.608687 | 0.00158 |
| Spinocerebellar ataxia | KEGG:05017 | 12 | 3.541735 | 0.00235 |
| Cardiac muscle contraction | KEGG:04260 | 8 | 3.853842 | 0.016557 |
| Carbon metabolism | KEGG:01200 | 9 | 3.223887 | 0.02722 |

**Supplementary Table 24.**  KEGG pathways enriched in the genes common to both the PFC_M23 and the Torin1_M6 modules.

| **Term_name** | **Term_id** | **Count** | **Fold Enrichment** | **Adjusted_p_value** |
| --- | --- | --- | --- | --- |
| mTOR signaling pathway | KEGG:04150 | 11 | 5.218567 | 0.0018 |
| Epithelial cell signaling in Helicobacter pylori infection | KEGG:05120 | 7 | 7.211111 | 0.0057 |
| Synaptic vesicle cycle | KEGG:04721 | 7 | 6.47151 | 0.0063 |
| Autophagy - animal | KEGG:04140 | 9 | 4.772059 | 0.0063 |
| Oocyte meiosis | KEGG:04114 | 8 | 4.542432 | 0.0166 |
| Collecting duct acid secretion | KEGG:04966 | 4 | 10.68313 | 0.0166 |
| Dopaminergic synapse | KEGG:04728 | 8 | 4.37037 | 0.0166 |
| Alzheimer disease | KEGG:05010 | 9 | 3.795322 | 0.0176 |

**Supplementary Table 25.** Co-expression modules from the brain endothelial cells of LPS-treated mice as compared to wild-type mice

| Module | LPS treatment |
| --- | --- |
| Endo_LPS_M1 | N.S |
| Endo_LPS_M2 | N.S |
| Endo_LPS_M3 | N.S |
| Endo_LPS_M4 | N.S |
| Endo_LPS_M5 | N.S |
| Endo_LPS_M6 | N.S |
| Endo_LPS_M7 | 0.95 |
| Endo_LPS_M8 | N.S |

N.S, not significant; adjusted p values less than 0.05 were considered significant.

**Supplementary Table 26.**  Consensus genes between the Endo_LPS_M7 module from the brain endothelial cells of LPS-treated mice and the differentially expressed genes

| Module | Down regulated genes (n=1759) | Up regulated genes (n=1301) |
| --- | --- | --- |
| Endo_LPS_M7 | 310 (P=0.86) | 678 (P<2.2e-16) |

**Supplementary Table 27.**  KEGG pathways enriched in the genes common to both the PFC_M4 and the Endo_LPS_M7 modules.

| **Term_name** | **Term_id** | **Count** | **Fold Enrichment** | **Adjusted p_value** |
| --- | --- | --- | --- | --- |
| TNF signaling pathway | KEGG:04668 | 9 | 14.6 | 1.19E-06 |
| NF-kappa B signaling pathway | KEGG:04064 | 6 | 10.7 | 4.30E-04 |
| Adipocytokine signaling pathway | KEGG:04920 | 4 | 10.6 | 5.04E-03 |
| Kaposi sarcoma-associated herpesvirus infection | KEGG:05167 | 10 | 9.4 | 5.72E-06 |
| Hepatitis C | KEGG:05160 | 8 | 9.3 | 7.90E-05 |
| AGE-RAGE signaling pathway in diabetic complications | KEGG:04933 | 5 | 9.1 | 2.86E-03 |
| Osteoclast differentiation | KEGG:04380 | 6 | 8.7 | 1.06E-03 |
| Influenza A | KEGG:05164 | 8 | 8.7 | 1.01E-04 |
| Insulin resistance | KEGG:04931 | 5 | 8.4 | 3.06E-03 |
| HIF-1 signaling pathway | KEGG:04066 | 5 | 8.4 | 3.06E-03 |
| Toxoplasmosis | KEGG:05145 | 5 | 8.4 | 3.06E-03 |
| PD-L1 expression and PD-1 checkpoint pathway in cancer | KEGG:05235 | 4 | 8.2 | 9.46E-03 |
| Acute myeloid leukemia | KEGG:05221 | 3 | 8.2 | 3.02E-02 |
| Small cell lung cancer | KEGG:05222 | 4 | 7.9 | 1.03E-02 |
| Prolactin signaling pathway | KEGG:04917 | 3 | 7.8 | 3.31E-02 |
| Lipid and atherosclerosis | KEGG:05417 | 9 | 7.7 | 7.90E-05 |
| Non-small cell lung cancer | KEGG:05223 | 3 | 7.6 | 3.48E-02 |
| Epstein-Barr virus infection | KEGG:05169 | 8 | 7.4 | 2.85E-04 |
| Chronic myeloid leukemia | KEGG:05220 | 3 | 7.2 | 3.72E-02 |
| Pancreatic cancer | KEGG:05212 | 3 | 7.2 | 3.72E-02 |
| Toll-like receptor signaling pathway | KEGG:04620 | 4 | 7.1 | 1.40E-02 |
| Th17 cell differentiation | KEGG:04659 | 4 | 6.9 | 1.50E-02 |
| Hepatitis B | KEGG:05161 | 6 | 6.8 | 2.86E-03 |
| JAK-STAT signaling pathway | KEGG:04630 | 6 | 6.7 | 2.86E-03 |
| Apoptosis | KEGG:04210 | 5 | 6.7 | 6.94E-03 |
| Fluid shear stress and atherosclerosis | KEGG:05418 | 5 | 6.6 | 7.30E-03 |
| Salmonella infection | KEGG:05132 | 8 | 5.9 | 1.06E-03 |
| Necroptosis | KEGG:04217 | 5 | 5.7 | 1.07E-02 |
| Pathogenic Escherichia coli infection | KEGG:05130 | 6 | 5.6 | 5.84E-03 |
| FoxO signaling pathway | KEGG:04068 | 4 | 5.6 | 3.00E-02 |
| Human T-cell leukemia virus 1 infection | KEGG:05166 | 6 | 5.0 | 9.01E-03 |
| MAPK signaling pathway | KEGG:04010 | 8 | 5.0 | 2.61E-03 |
| Human cytomegalovirus infection | KEGG:05163 | 6 | 4.9 | 9.46E-03 |
| PI3K-Akt signaling pathway | KEGG:04151 | 9 | 4.6 | 1.75E-03 |
| Proteoglycans in cancer | KEGG:05205 | 5 | 4.4 | 2.89E-02 |
| Rap1 signaling pathway | KEGG:04015 | 5 | 4.3 | 3.00E-02 |
| MicroRNAs in cancer | KEGG:05206 | 7 | 4.1 | 9.46E-03 |
| Shigellosis | KEGG:05131 | 5 | 3.7 | 4.68E-02 |
| Human papillomavirus infection | KEGG:05165 | 6 | 3.3 | 3.92E-02 |
| Pathways in cancer | KEGG:05200 | 8 | 2.8 | 3.50E-02 |

**Supplementary Table 28.**  KEGG pathways enriched in the genes common to both PFC_M5 and the Endo_LSP_M7 modules.

| **Term_name** | **Term_id** | **Count** | **Fold Enrichment** | **Adjusted p_value** |
| --- | --- | --- | --- | --- |
| Ribosome | KEGG:03010 | 48 | 19.5 | 4.57E-49 |
| Coronavirus disease - COVID-19 | KEGG:05171 | 50 | 13.4 | 1.22E-42 |
| Regulation of actin cytoskeleton | KEGG:04810 | 12 | 3.5 | 1.27E-02 |

**Supplementary Table 29.**  Summary of the results from this study.

|  | **Up-regulation of mTOR pathway in the PFC of BPD** | **Down-regulation of mTOR pathway in the PFC of BPD** |
| --- | --- | --- |
| **Discovery study**  Differential gene expression analysis | mTOR related pathway (ribosome) enriched in the up-regulated genes  3 genes involving in mTOR pathway in the up-regulated genes | mTOR related pathway (autophagy) enriched in the down-regulated genes  15 genes involving in mTOR pathway in the down-regulated genes |
|  |  |  |
| **Discovery study**  Network analysis | mTOR related signaling pathways enriched in the upregulated modules, PFC_M4* and PFC_M5* | mTOR and related pathways enriched in the downregulated module, PFC_M23^#^ |
|  |  |  |
| **Discovery study**  Cell type enrichment analysis | Endothelial cell markers enriched in the PFC_M4  No specific cell marker genes enriched in the PFC_M5 | Neuronal markers enriched in PFC_M23 |
|  |  |  |
| **Discovery study**  Transcription factor analysis | STAT3 target genes enriched in the PFC_M4 | No transcription factors regulated by mTORC1 identified in the PFC_M23 |
|  | p53 target genes enriched in the PFC_M5 |  |
|  |  |  |
| **Replication study**  Differential gene expression analysis | Pathways related to mTOR enriched in the commonly up-regulated genes  Replicated 1/3 up-regulated genes involved in mTOR pathway | mTOR pathway and mTOR related pathways enriched in the commonly down-regulated genes  Replicated 10/15 down-regulated genes involved in mTOR pathway |
|  |  |  |
| **Replication study**  Network analysis | R_PFC_M18* overlapped significantly with PFC_M4 | R_PFC_M3^#^ overlapped significantly with PFC_M23 |
|  | No replication modules overlapped with PFC_M5 |  |
|  | mTOR related signaling pathways were enriched in the genes common to both PFC_M4 and R_PFC_M18 | mTOR and related pathways were enriched in the genes common to both PFC_M23 and R_PFC_M3 |
|  |  |  |
|  |  |  |
| **Torin1 treated neurons** |  | PFC_M23 significantly overlapped with the Torin1_M6* |
| **Brain endothelial cells from LPS-treated mice** | The PFC_M4 and PFC_M5 overlapped with the Endo_LPS_M7^*^ | No overlapped modules |
|  |  |  |
|  | mTOR related pathways enriched in the common genes between the PFC_M4 and Endo_LPS_M7 |  |
|  |  |  |
|  | mTOR related pathways enriched in the common genes between the PFC_M5 and Endo_LPS_M7 |  |

*Up-regulated modules in the PFC of BPD, in the neurons by Torin1 treatment or in the brain endothelial cells from LPS-treated mice; PFC_M4, PFC_M5, R_PFC_M18, Endo_LPS_M7

^#^Down-regulated modules in the PFC of BPD or in the neurons by Torin1 treatment; PFC_M23, R_PFC_M3, Torin1_M6

Data that did not validate the discovery study are in red.
