# Supplementary material for: Differential expression of gene co-expression networks related to the mTOR signaling pathway in bipolar disorder
Source: Transl Psychiatry. 2022 May 4;12:184. doi: 10.1038/s41398-022-01944-8 (PMC9067344; doi:10.1038/s41398-022-01944-8)
Supplement: Supplementary file 3 — Supplementary Methods [file 41398_2022_1944_MOESM3_ESM.docx]

**Supplementary Methods**

**Western blot analysis**

Western blot analysis was performed as previously described ^1^. Equal quantities of protein from cell lysates were separated by SDS-PAGE and transferred to polyvinylidene fluoride membranes. Membranes were blocked with 5% non-fat milk in Tris-buffered saline (TBS) with 0.15% Tween 20 (TBS-T) and then incubated with phospho-mTORC1 (Ser2448, #2971), phospho-Akt (Ser473, #2971), phospho-S6K (Thr389, #9205), phospho-S6 (Ser240/244, #2215), LC3B (#2775), Beclin 1 (#3738), PSD-95 (#3450), GluA 1 (#13185) (1:1000; Cell Signaling, Danvers,. MA, USA) or α-tubulin (1:2000; T9026, Sigma, St. Louis, MO, USA) antibodies at 4°C overnight. Membranes were then washed three times in TBS-T for 10 min and incubated for 1 h in TBS-T containing the horseradish peroxidase-conjugated secondary antibody [goat-anti-rabbit IgG (Thermo Fisher Scientific) for anti-phospho-mTORC1, anti-phospho-Akt, anti-phospho-S6K, anti-phospho-S6, anti-PSD-95, anti-GluA1, anti-Beclin 1, and anti-LC3B (1:1000); and anti-mouse IgG (Sigma) for anti-α-tubulin (1:10000)]. After detection of phosphorylated proteins, membranes were incubated in stripping buffer [25 mM glycine (pH 2.0) and 2% SDS], and blocked with 5% non-fat milk in TBS-T. Membranes were reprobed with total mTORC1 (#2972), total Akt (#9272), total S6K (#9202) or total S6 (#2217) (1:1000; Cell Signaling) overnight. Immunoreactive bands were analyzed using enhanced chemiluminescence (ECL) detected in the Amersham Imager 600 imaging system (GE Healthcare Life Sciences, Chicago, IL, USA). Data are presented as the means ± standard error of the mean (SEM) of two to three independent experiments in duplicate wells (n = 4-6).

**Cell Viability (MTT) assay**

Cytotoxicity effects of Torin 1 and LY2584702 on neuronal cells were determined respectively with 3-[4,5-dimethylthiazol-2-yl]-2,5-diphenyltetrazolium bromide (MTT) assay. Cells were cultured with drugs for 24 h in 96-well plate prior to the addition of MTT. The plates were incubated at 37°C for 4 h to dissolve the formazan that had formed using ELISA reader (Spectramax M2e; Molecular Devices, San Jose, CA, USA). The data were expressed as percentage of the control, and experiment was carried out in triplicate (n =3).

**Neurite assay**

Dendrites were visualized via immunostaining using a microtubule-associated protein 2 antibody (MAP2; MAB3418, Millipore, Temecula, CA, USA), which is a dendritic marker. Cortical cells were incubated with anti-MAP2 antibody diluted 1/200. Alexa Fluor 568 goat anti-mouse IgG (A11031, Invitrogen, Carlsbad, CA, USA) was used as a secondary antibody and Hoechst 33258 (H21491, Invitrogen) was used for nuclear staining. To analyze total dendritic length, five fields were randomly selected from each group. All neurons in a given field were counted, including both basal and apical dendrites, and dendritic length was determined to be the distance between the edge of the cell body and the tip of the growth cone. Total dendritic length was obtained by summing the lengths of all dendrites from a single neuron and then averaging this measure in each group using MetaMorph (Molecular Devices, Downingtown, PA, USA), an automated image-analysis program. At least 100 cells were analyzed in 5 fields by a researcher blind to the groups.

**Spine density assay**

Spines were immunostained with Alexa Fluor® 488 phalloidin (Molecular Probe, Eugene, OR, USA). To analyze spine density, spines and filopodia were differentiated by shape and length such that spines were defined as less than 3 µm long with a rounder or mushroom shape while filopodia were defined as between 3 and 10 µm long with a narrower shape. Ten neurons were randomly selected from each group. Using 10 neurons per group, two dendritic segments per neuron (50 µm) were analyzed (20 dendritic segments per group) by a researcher blind to the groups. To represent average spine density in a 10-µm dendrite, the spine density of a 50-µm dendritic segment was divided by 5.

**Statistical analysis**

All statistical analyses were performed using GraphPad Prism software (ver. 8.1, USA). Dose response studies were analyzed using one-way analysis of variance (ANOVA), followed by *post hoc* Tukey’s multiple comparisons. Data for control vs. treatment group was analyzed using *unpaired Student* *t*-*test*. *P*-values < 0.05 were considered significant.

1. Park SW, Lee JG, Seo MK, Lee CH, Cho HY, Lee BJ *et al.* Differential effects of antidepressant drugs on mTOR signalling in rat hippocampal neurons. *Int J Neuropsychopharmacol* 2014; **17**(11)**:** 1831-1846.
